# Absence of 3*a*_0_ charge density wave order in the infinite-layer nickelate NdNiO_2_

**DOI:** 10.1038/s41563-024-01797-0

**Published:** 2024-01-26

**Authors:** C. T. Parzyck, N. K. Gupta, Y. Wu, V. Anil, L. Bhatt, M. Bouliane, R. Gong, B. Z. Gregory, A. Luo, R. Sutarto, F. He, Y.-D. Chuang, T. Zhou, G. Herranz, L. F. Kourkoutis, A. Singer, D. G. Schlom, D. G. Hawthorn, K. M. Shen

**Affiliations:** 1 Laboratory of Atomic and Solid State Physics, Department of Physics, Cornell University, Ithaca, NY USA; 2 Department of Physics and Astronomy, University of Waterloo, Waterloo, Ontario Canada; 3 School of Applied and Engineering Physics, Cornell University, Ithaca, NY USA; 4 Department of Materials Science and Engineering, Cornell University, Ithaca, NY USA; 5 Canadian Light Source, Saskatoon, Saskatchewan Canada; 6 Advanced Light Source, Lawrence Berkeley National Laboratory, Berkeley, CA USA; 7 Center for Nanoscale Materials, Argonne National Laboratory, Lemont, IL USA; 8 Institut de Ciència de Materials de Barcelona (ICMAB-CSIC), Bellaterra, Spain; 9 Kavli Institute at Cornell for Nanoscale Science, Cornell University, Ithaca, NY USA; 10 Leibniz-Institut für Kristallzüchtung, Berlin, Germany

**Keywords:** Superconducting properties and materials, Electronic properties and materials

## Abstract

A hallmark of many unconventional superconductors is the presence of many-body interactions that give rise to broken-symmetry states intertwined with superconductivity. Recent resonant soft X-ray scattering experiments report commensurate 3*a*_0_ charge density wave order in infinite-layer nickelates, which has important implications regarding the universal interplay between charge order and superconductivity in both cuprates and nickelates. Here we present X-ray scattering and spectroscopy measurements on a series of NdNiO_2+*x*_ samples, which reveal that the signatures of charge density wave order are absent in fully reduced, single-phase NdNiO_2_. The 3*a*_0_ superlattice peak instead originates from a partially reduced impurity phase where excess apical oxygens form ordered rows with three-unit-cell periodicity. The absence of any observable charge density wave order in NdNiO_2_ highlights a crucial difference between the phase diagrams of cuprate and nickelate superconductors.

## Abstract (web summary)

Samples of NdNiO_2+*x*_ are prepared with meticulous control of the oxygen content. Their X-ray scattering and spectroscopy measurements then reveal that the previously reported charge density wave in undoped infinite-layer nickelates is, in fact, a spurious effect.

## Main

The discovery of superconductivity in infinite-layer nickelates^1^, and its analogy to its cuprate antecedents, offers a unique opportunity to better understand the key ingredients for high-temperature superconductivity. Although nickelates and cuprates share many commonalities, including a broadly similar crystal and electronic structure^1^, strong correlations, antiferromagnetic excitations^2,3^ and a superconducting dome^4–6^, there are also many distinctions between the two families. These include the very different transition temperatures^1,4,5^, the relative oxygen and 3*d* character of the doped holes^7,8^, and the hybridization between the 3*d* and rare-earth states^9^. One important apparent similarity between the two families is the report of charge density wave order in a variety of infinite-layer nickelates by resonant soft X-ray scattering (RSXS)^10–13^. This discovery, if correct, would suggest a ubiquitous interplay between charge order and superconductivity in the phase diagram of both cuprates and nickelates, with important implications for a universal theory of high-temperature superconductivity.

Nevertheless, there are clear distinctions between the charge order reported in cuprates and nickelates. In cuprates, the wavevector is typically incommensurate and strongly doping dependent^14–16^, whereas in NdNiO_2_ and PrNiO_2_, it is locked to **q** = (1/3, 0) (refs. ^11–13^). Additionally, charge ordering in cuprates is strongly temperature dependent^15–20^, whereas reports in nickelates exhibit a weak temperature dependence with no clear transition or onset^10,12,13^. To better understand the nature of putative charge ordering in the infinite-layer nickelates, we have investigated the **q** = (1/3, 0) superlattice peak in a series of samples with varying levels of reduction. We discover that the superlattice peak is entirely absent in fully reduced, single-phase NdNiO_2_ samples, and instead arises from partially reduced impurity phases, namely, NdNiO_2.33_ (Nd_3_Ni_3_O_7_) or NdNiO_2.67_ (Nd_3_Ni_3_O_8_), produced during the reduction process, where excess apical oxygen atoms form ordered rows with three-unit-cell periodicity. This reveals that charge ordering with 3*a*_0_ periodicity is not intrinsic to the infinite-layer nickelates—a discovery with important implications for our understanding of the phase diagram of nickelates and its relationship to cuprates.

To produce a sequence of NdNiO_2_ samples with nominally identical Nd:Ni stoichiometry^21^ and variable oxygen content, we have employed a combination of reactive-oxide molecular-beam epitaxy to synthesize the precursor perovskite and atomic hydrogen reduction to access the oxygen-deficient phases. A consistent series of perovskite films of NdNiO_3_ (20 pseudocubic unit cells thick) with excellent crystallinity and sharp metal–insulator transitions were synthesized on SrTiO_3_. Following synthesis, all the films were capped by 2–3 unit cells of SrTiO_3_ and exposed to a beam of >50% atomic hydrogen produced by a thermal cracker^22^. Although the reduction of perovskite nickelates has typically been achieved using CaH_2_ and NaH powder^1,5,23^, atomic hydrogen offers the benefit of independent control over the sample temperature and reducing environment, as well as fast reaction times (<20 min).

**Fig. 1:**
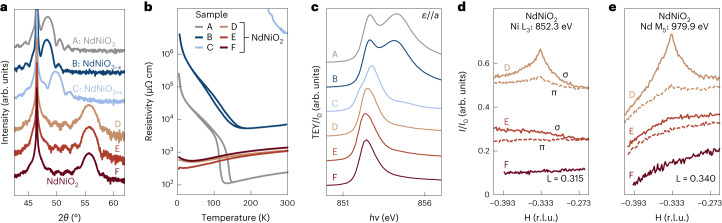
Characterization of perovskite NdNiO_3_, oxygen-deficient NdNiO_2+*x*_ and infinite-layer NdNiO_2_. **a**, Cu Kα *θ*–2*θ* XRD measurements of a sequence of thin films ranging from NdNiO_3_ (sample A) to partially reduced NdNiO_2+*x*_ (samples B and C) and NdNiO_2_ (samples D–F). **b**, Electrical transport measurements of the same series of samples. **c**, Total-electron-yield (TEY) XAS measurements of this sample series taken at the Ni L_3_ edge with the electric field *ε* parallel to the sample *a* axis. **d**,**e**, RSXS measurements of NdNiO_2_ samples D–F; rocking curves through the nominal charge order position of **q** = (–1/3, 0) at the Ni L_3_ (**d**) and Nd M_5_ (**e**) edges at σ and π polarizations, respectively. The plotted intensity is the measured scattered intensity *I* divided by the incident-beam intensity *I*_0_, without any background subtraction. Traces in **a** and **c**–**e** have been vertically offset for clarity. Source data

Using this approach, we have synthesized a series of samples ranging from the pristine parent perovskite NdNiO_3_ (sample A) to oxygen-deficient intermediate phases NdNiO_2+*x*_ (samples B and C) and infinite-layer NdNiO_2_ (samples D–I), all of which were characterized by conventional X-ray diffraction (XRD) and transport measurements. All the NdNiO_2_ samples appear highly crystalline and single phase by XRD, with low-temperature resistivities of 350–700 μΩ cm, comparable with or lower than undoped films on SrTiO_3_ (refs. ^4,5,24^) and LSAT^25^ (Fig. 1a,b; Supplementary Information shows the data for samples G–I). Further information about the growth and reduction procedures are available in Methods.

X-ray absorption spectroscopy (XAS) measurements on all the samples are shown in Fig. 1c, and RSXS measurements on three representative infinite-layer NdNiO_2_ samples (samples D–F) are presented in Fig. 1d,e. The XAS spectra of all three NdNiO_2_ samples appear similar and closely match with the published measurements^9,26^, with a single peak at the Ni L_3_ edge (852.4 eV) with no visible prepeak at the O K edge. Despite the apparent similarity between these samples, only sample D exhibits a superlattice peak at the putative charge order wavevector of **q** = (1/3, 0). As shown in previous reports, this peak is observed on the Ni L edge (Fig. 1d) as well as the Nd (rare-earth) M edge (Fig. 1e), and exhibits a strong polarization dependence, namely, *I*_σ_/*I*_π_ ≈ 4 at *hν* = 852.3 eV, consistent with prior measurements^11,13^. The estimated in-plane correlation length of *ξ* = 12–20 nm is also qualitatively similar to earlier reports^10–13^. Finally, a weak dependence on the out-of-plane momentum transfer L is observed with a maximum at roughly L = 0.31(2) reciprocal lattice units (r.l.u.), again consistent with earlier measurements (all the momenta in this text are quoted in r.l.u. with reference to the NdNiO_2_ lattice; *a* = 3.905 Å, *c* = 3.286 Å). In contrast, the superlattice peak was not observed in samples E and F, which is notable since sample E exhibited the lowest resistivity of all the samples. Additional measurements on samples G and H were measured at a separate beamline and exhibited an extremely weak **q** = (1/3, 0) feature at the sample centre, whereas sample I showed no superlattice features whatsoever. Supplementary Information provides further details of the measurements on samples E–I.

This discrepancy across nominally similar NdNiO_2_ samples suggests that some unknown variable, potentially associated with sample disorder or oxygen non-stoichiometry, influences the presence of the 3*a*_0_ superlattice peak. One possibility is that the charge density wave order is intrinsic to infinite-layer nickelates, but triggered by the presence of atomic-scale disorder within the lattice. Alternatively, the peak could originate from an impurity phase produced during the reduction process, which would imply that the charge order observed to date does not play a role in the phase diagram of infinite-layer nickelates.

To distinguish between these scenarios, we have investigated partially reduced, oxygen-deficient perovskite phases NdNiO_2+*x*_ (samples B and C). Sample B was only lightly reduced, with its XRD data nearly indistinguishable from the perovskite (sample A), but with a substantially broadened metal–insulator transition and increased sample resistance (Fig. 1a,b). Sample C was reduced further and shows an intermediate out-of-plane lattice constant of *c* = 3.66 Å and highly insulating electrical transport as expected for a predominantly oxygen-deficient perovskite phase^27^. The XAS spectra of sample B exhibit the typical two-peak structure observed in perovskite nickelates on the Ni L_3_ edge^28^, as well as a strong prepeak at the O K edge; sample C is markedly different, with a sharp peak at 852.7 eV, shoulder at 852.0 eV and weak secondary peak at 854.3 eV, indicative of an oxygen-deficient perovskite phase^29,30^.

**Fig. 2:**
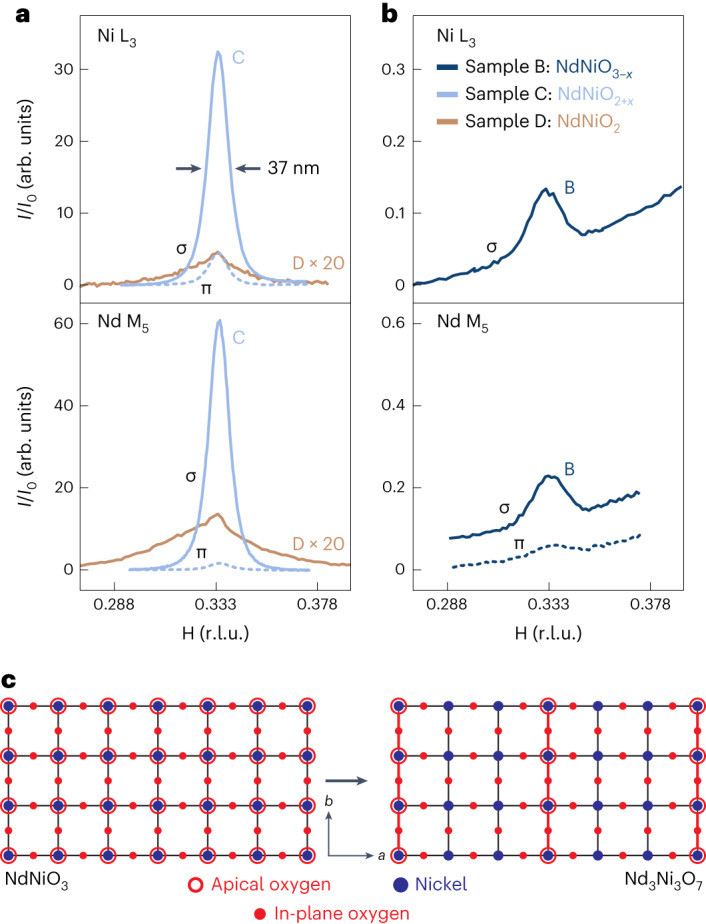
X-ray scattering measurements of oxygen-deficient perovskite, NdNiO_2+*x*_, samples B and C. **a**, H scans, at constant L, at the Ni L_3_ and Nd M_5_ edges for sample C are presented without background subtraction. Data for sample D, after removal of a cubic fluorescent background and rescaling for visibility, are included for reference. **b**, Rocking curves through **q** = (1/3, 0) on lightly reduced sample B, presented without background subtraction but vertically offset for clarity. **c**, Illustration of a potential intermediate-phase structure for Nd_3_Ni_3_O_7_ from a top-down view of the *a*–*b* plane of NiO_2_ where two-thirds of the rows of apical oxygen atoms are removed, forming a 3*a*_0_ supercell along the *a* axis. Source data

In Fig. 2a, we show the RSXS measurements of sample C, which exhibit a peak at **q** = (1/3, 0) virtually identical to the superlattice peak in sample D and the literature^10–13^ in nearly all respects: wavevector, energy dependence, polarization dependence, temperature dependence and correlation length, with the exception that it is extremely intense (100–400 times stronger than that observed in sample D). This peak also displays a strong L dependence, with a maximum at 0.30 r.l.u (*d*/3 = 3.65 Å). The strong similarity between the superlattice features in samples C and D suggests a common origin: ordered oxygen-deficient phases arising from an incomplete reduction process. In reduced nickelates, excess apical oxygens can form ordered phases, such as the brownmillerite structure of La_2_Ni_2_O_5_ (refs. ^31–33^), where the apical oxygen atoms form alternating rows. Other related structures also exist with different periodicities, such as Nd_3_Ni_3_O_7_ (refs. ^34–36^), Pr_3_Ni_3_O_7_ (refs. ^34,35^), La_3_Ni_3_O_8_ (ref. ^37^) and (Pr,Ca)_4_Ni_4_O_11_ (ref. ^38^). Recent in situ XRD studies indicate that the reduction pathway from bulk NdNiO_3_ to NdNiO_2_ occurs first via the formation of an intermediate-phase Nd_3_Ni_3_O_7_ (ref. ^36^), where one-third of the apical oxygen sites are occupied and are ordered into chains with 3*a*_0_ periodicity (Fig. 2c). This 3 × 1 × 3 superstructure of the original pseudocubic unit cell would naturally give rise to a superlattice peak at **q** = (1/3, 0, 1/3). This suggests that sample C is probably predominantly Nd_3_Ni_3_O_7_, and that an incomplete reduction of any NdNiO_2_ samples would also leave traces of the Nd_3_Ni_3_O_7_ phase behind (sample D).

To further investigate this hypothesis, we have also performed RSXS measurements on sample B, which should only be in the initial stages of the conversion from NdNiO_3_ into a brownmillerite-like Nd_3_Ni_3_O_7_ phase. The XRD and XAS measurements of this sample (Fig. 1a,c) are indistinguishable from the data for pristine NdNiO_3_ (sample A). Nevertheless, RSXS measurements (Fig. 2b) also show the **q** = (1/3, 0) peak with identical characteristics (L, energy and polarization dependence) and comparable intensity with sample D. The fact that sample B exhibits no obvious trace of the infinite-layer phase and still exhibits a clear **q** = (1/3, 0) superlattice peak demonstrates that this feature does not originate from intrinsic charge ordering within the infinite-layer phase itself. In fact, the **q** = (1/3, 0) peaks appear to be more reminiscent of Bragg peaks observed in resonant scattering from cuprates with oxygen ordering (for example, ortho-YBa_2_Cu_3_O_6+*δ*_), as opposed to intrinsic charge density wave order^39,40^. There, the strong resonant enhancement on the Cu L edge arises from the local oxygen environment strongly modifying the electronic structure of the Cu atoms, and a similar resonant enhancement at the Ni L edge should likewise occur for oxygen ordering in nickelates.

**Fig. 3:**
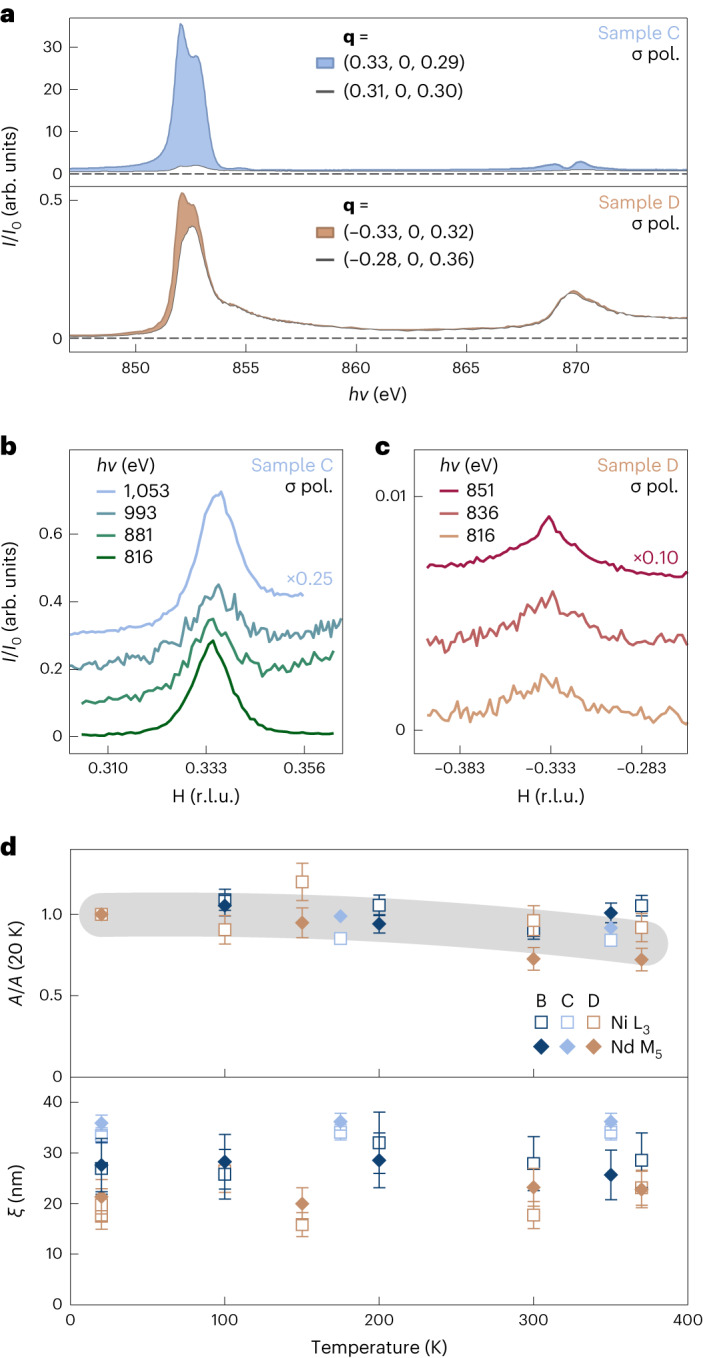
Energy and temperature dependence of the **q** = (1/3, 0) scattering peak. **a**, Fixed-wavevector resonant energy profiles both on and off the scattering peak for samples C and D. The shaded region indicates intensity attributable to the resonant scattering above the fluorescent background (white). **b**,**c**, Rocking curves through **q** = (1/3, 0) for a wide range of photon energies around the Ni L and Nd M resonances for sample C (**b**) and sample D (**c**); traces have been vertically offset for clarity, but no background subtraction has been applied. **d**, Temperature dependence of peak amplitude *A* and correlation length *ξ* = 2π/Δ*q*, at both edges for samples B–D. The error bars represent the fitting uncertainty in the extracted quantities (*A* and *ξ*); they define the range of values obtained by fitting the RSXS data with different models of the fluorescence background (that is, different polynomial degrees and fits over different ranges). The grey line is a guide to the eye. Source data

In Fig. 3a, we show the energy dependence of the **q** = (1/3, 0, 1/3) peak intensity in samples C and D (shaded), together with the background fluorescence measured off the superlattice peak (white). Because the superlattice peak intensity is far stronger in sample C, the relative strength of the background fluorescence in sample D is much larger than in sample C. Nevertheless, a two-peak resonance profile is apparent in both samples at the Ni L_3_ edge with only a weak response at the L_2_ edge, similar to prior measurements on NdNiO_2_ (ref. ^11^). Assuming a structural origin arising from oxygen ordering, the superlattice peak should also be observable in the off-resonance case. In Fig. 3b,c, we show a series of scans across **q** = (1/3, 0) for samples C and D, spanning a 235 eV range about the Ni and Nd resonances. Although the intensity is weaker than the on-resonance case, the persistence of the peak strongly supports a structural origin. The temperature dependence of the scattering peak is shown for samples B–D (Fig. 3d). Similar to prior reports^10,12,13^, the peak shows a smooth decrease in intensity by 15–20% between 20 and 370 K, with no obvious change in the correlation length. This weak dependence—without a transition—is fairly similar to the temperature dependence of superlattice peaks resulting from the oxygen ordering in YBa_2_Cu_3_O_6+*δ*_ (refs. ^40,41^), as opposed to the more dramatic temperature dependence of the CDW order^18,40^.

We also observe a strong dependence of the **q** = (1/3, 0) peak intensity in sample G for the measurement location on the 10 × 10 mm^2^ sample (Supplementary Information). Due to the spread of the atomic hydrogen beam and thermal gradients, the edges of the sample are not as well reduced as the centre—correspondingly, we observe a clear superlattice peak near the edge of the sample, which decreases in intensity approaching the sample centre. Spatially resolved synchrotron hard XRD measurements at the same locations confirm the prevalence of intermediate reduction products at the edge of sample G, versus fully reduced NdNiO_2_ near the centre, thereby correlating the presence of the resonant feature with that of intermediate phases on the same sample.

**Fig. 4:**
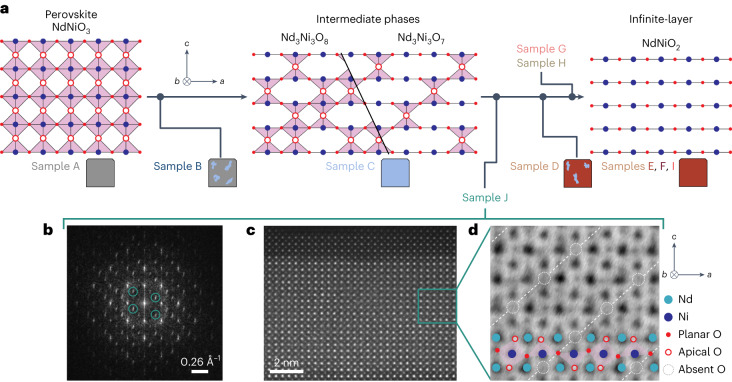
Outline of the multistage reduction process of nickelate films and high-resolution STEM images of a partially reduced film showing 3*a*_0_ ordering. **a**, Schematic of the reduction pathway from the perovskite NdNiO_3_ to the intermediate oxygen-deficient phases of Nd_3_Ni_3_O_7_ or Nd_3_Ni_3_O_8_ and the infinite-layer NdNiO_2_; the corresponding samples measured in this study that lie on this pathway are also shown. **b**–**d**, STEM images of a mixed-phase sample (sample J) containing Nd_3_Ni_3_O_7,8_ intermediate phases. Fourier transform image (**b**) and corresponding high-angle annular dark-field image (**c**) of a defective region are shown. The positions of the third-order peaks in the Fourier transform are circled in green. An annular bright-field image showing two filled rows of apical oxygens (**d**), followed by one row of missing apical oxygen positions, corresponding to the schematic for Nd_3_Ni_3_O_8_ in **a**. In this image, the apical oxygen chains run into the page. Source data

Prior powder XRD studies of bulk Nd_3_Ni_3_O_7_ reveal that the excess apical oxygens form octahedrally coordinated chains that run perpendicular to the NiO_2_ planes and which are separated by 3*a*_0_ (ref. ^34^). Another potential arrangement is to have the apical oxygens form pyramidal chains that lie within the NiO_2_ plane and are oriented along the *a* axis (Fig. 4a). In addition, Nd_3_Ni_3_O_8_ (NdNiO_2.67_), where two rows of apical oxygens are occupied followed by a row of vacancies, would likewise generate the same superlattice peak at **q** = (1/3, 0, 1/3), although this phase has not been previously reported. Scanning transmission electron microscopy (STEM) measurements on a separate, partially reduced sample (sample J) are detailed in Fig. 4b,c,d. The Fourier transform of a high-angle annular dark-field image reveals 1/3-order diffraction peaks corresponding to 3*a*_0_ ordering along both *a* and *c*, consistent with other recent transmission electron microscopy measurements on uncapped NdNiO_2_ films^42^. Additionally, annular bright-field data show regions with two filled apical oxygen chains alternating with a single vacant chain, consistent with the Nd_3_Ni_3_O_8_ structure shown in Fig. 4a, where the apical oxygen chains are directed into the page. Sample J was prepared using the same conditions as samples D–I, but with a four-unit-cell SrTiO_3_ cap. Although RSXS measurements were not performed on sample J, XRD and transport measurements (Supplementary Information) indicate that this sample is less reduced than sample D as both NdNiO_2_ and intermediate-phase peaks are visible in lab-based XRD. Since the difference in formation energies of the various ordered oxygen structures is small, it is possible that multiple compositions or structures of Nd_3_Ni_3_O_7,8_ could exist within our series of samples, depending on factors such as the reduction conditions or epitaxial strain^43^. A more detailed investigation of the precise structures of Nd_3_Ni_3_O_7_ or Nd_3_Ni_3_O_8_ in our thin films is currently the subject of further investigation. Nevertheless, the key point is that all the configurations of Nd_3_Ni_3_O_7_ or Nd_3_Ni_3_O_8_ will form a 3 × 1 × 3 supercell that will generate the **q** = (1/3, 0) superlattice peak at the observed wavevector. Finally, a macroscopic sample would be expected to possess equal domains of chains along both in-plane directions, in which case the superlattice peaks would then be observable along both H and K directions.

Our results suggest the scenario illustrated in Fig. 4a. The reduction of NdNiO_3_ into NdNiO_2_ occurs via the production of an intermediate, partially reduced Nd_3_Ni_3_O_7_ and/or Nd_3_Ni_3_O_8_ phase, where excess apical oxygen atoms (or vacancies) are ordered into rows with 3*a*_0_ periodicity forming a 3 × 1 × 3 supercell with a superlattice peak at the putative charge order wavevector of **q** = (1/3, 0, 1/3) (sample C). Because of its extremely strong intensity in the on-resonance case, this peak is detectable even if very small amounts of the partially reduced phase are present at levels nearly undetectable by conventional techniques (XRD, transport and XAS; that is, samples B, D and H). This picture is supported by a multitude of our observations: (1) the superlattice peak is absent in the plurality of low-resistivity, fully reduced NdNiO_2_ samples (samples E, F and I); (2) the superlattice peak from Nd_3_Ni_3_O_7,8_ (sample C) exhibits nearly identical energy, polarization and temperature dependence to NdNiO_2_ (sample D) as well as prior measurements on RENiO_2_ (refs. ^10–13^); (3) the peak is commensurate to the lattice and observable well off-resonance, indicating a strong structural component; (4) even a lightly reduced NdNiO_3_ sample devoid of any detectable NdNiO_2_ exhibits the superlattice peak (sample B); (5) position-dependent measurements correlate the **q** = (1/3, 0) peak, observed by RSXS, with the presence of partially reduced phases, observed by hard XRD at the same locations on the same sample (sample G); and (6) the direct observation of excess apical oxygen ordering with 3*a*_0_ periodicity in a partially reduced sample by STEM (sample J).

The presence of an ordered oxygen impurity phase also explains numerous discrepancies and unusual features in the literature. The strong intensity on the rare-earth edge is surprising for a charge ordering scenario, since the coupling of the rare-earth 4*f* electrons to the Ni3*d* electrons should be relatively weak and indirect, but should naturally occur for a structural Bragg peak where the Nd ions are displaced due to oxygen ordering. In addition, prior reports claim the presence/absence of a superlattice peak in uncapped versus capped samples of NdNiO_2_. Our measurements indicate that although the capping layer alone does not dictate the presence of the superlattice peak (all the samples were capped), even small variations in reduction conditions can result in residual amounts of Nd_3_Ni_3_O_7,8_. The doping dependence of the intensity of the superlattice peak, which is the strongest for the undoped parent compound and vanishing near *x* = 0.20, may also be naturally explained by the presence of an excess oxygen impurity phase. Bulk studies indicate that Sr doping, which increases the average targeted Ni valence of RE_1−*x*_Sr_*x*_NiO_2_, makes the material easier to reduce^36^. Thus, the addition of Sr may naturally diminish the amount of residual RE_3_Ni_3_O_7,8_ in a sample and suppress the intensity of the **q** = (1/3, 0) Bragg peak. Finally, although our study is limited to NdNiO_2+*x*_, these findings should broadly apply to all infinite-layer nickelates, since Pr_3_Ni_3_O_7_ and La_3_Ni_3_O_8_ also form 3 × 1 × 3 superstructures and are known to be the reduction products of PrNiO_3_ and LaNiO_3_, respectively^34,35,37^. Although the superlattice peak in LaNiO_2_ shares many overall similarities with those in (Nd,Pr)NiO_2_, including its energy and temperature dependence, it does exhibit some subtle apparent differences, particularly a very slight displacement from the commensurate **q** = (1/3, 0) wavevector (Δ*q* ≈ 0.01 r.l.u) (ref. ^10^) at *x* = 0. Future experiments will be important for conclusively determining the origins of the superlattice peak in the other members of infinite-layer nickelates. Although we focus here specifically on ordered excess oxygen phases, this highlights the role that excess oxygen may play in reduced films more broadly. The ordered excess oxygen phases appear to be highly insulating, and therefore, small inclusions may not substantially contribute to the electrical properties as a whole when averaging over an entire macroscopic sample. Nevertheless, if low concentrations of excess apical oxygen ions are randomly distributed, it is conceivable that they could potentially act as hole dopants, which could be consistent with traces of superconductivity observed in nominally undoped LaNiO_2_ (ref. ^6^).

Through a multimodal investigation of a large sequence of samples with varying levels of reduction, we conclude that charge ordering with 3*a*_0_ periodicity is not intrinsic to the infinite-layer nickelates. The superlattice peak previously identified as charge ordering at **q** = (1/3, 0) originates from the three-unit-cell ordering of excess apical oxygen ions in small amounts of brownmillerite-like inclusions of Nd_3_Ni_3_O_7_ or Nd_3_Ni_3_O_8_ produced during the reduction process. Topotactically reduced complex oxides present an exciting new frontier in quantum materials^1,44^, but this work also highlights some of the materials challenges inherent in these systems. We demonstrate RSXS as a highly sensitive and powerful probe for investigating these reduced compounds, which can detect even trace amounts of impurity phases. Although the phase diagrams of nickelates and cuprates show many similarities, including a superconducting dome and strong antiferromagnetic fluctuations on the underdoped side, this work establishes a clear distinction between the two families, namely, the fact that charge ordering does not appear to be directly relevant to the phase diagram of nickelates. This finding should have important implications for understanding universal models of high-temperature superconductivity, and may help to explain some of the key differences between the two families of materials.

## Methods

### Sample synthesis and characterization

Thin films of NdNiO_3_ were grown on SrTiO_3_(100) substrates using reactive-oxide molecular-beam epitaxy in a Veeco GEN10 system using elemental beams of Nd (Alfa Aesar, 99.900%) and Ni (Alfa Aesar, 99.995%). Substrates were etched to prepare a TiO_2_-terminated surface^45^ and annealed before growth at 650 °C until a clear reflection high-energy electron diffraction pattern was observed. Growths were performed at substrate temperatures between 480 and 500 °C in background pressures between 2 and 6 × 10^−6^ torr of ~80% distilled ozone. Initial flux calibration was performed by monitoring the reflection high-energy electron diffraction oscillations during the growths of binary oxides Nd_2_O_3_ on (ZrO_2_)_0.905_(Y_2_O_3_)_0.095_(111) and NiO on MgO(100) using the parameters outlined elsewhere^46^. Further stoichiometric optimization was performed by minimizing the NdNiO_3_(002)_pc_ plane spacing^21^. Following the growth of the nickelate layer, a SrTiO_3_ capping layer was grown at 500 °C in a background pressure of 2 × 10^−6^ torr, following the calibration of the Sr and Ti sources by monitoring the reflection high-energy electron diffraction oscillations.

Samples were reduced using a beam of atomic hydrogen produced by a thermal source^22,47^, where molecular H_2_ is passed through a heated tungsten capillary (>1,900 °C) where it disassociates into individual atoms before interacting with the sample. Reductions were performed in an ultrahigh-vacuum chamber (*P*_base_ < 1 × 10^−10^ torr) located on the same vacuum manifold as the molecular-beam epitaxy growth system at temperatures between 250 and 310 °C and hydrogen fluxes ranging between 1.8 and 2.7 × 10^15^ atoms cm^–2^ s^–1^. Typical reductions involved between 12 and 15 min of exposure to the atomic hydrogen beam to produce samples appearing fully reduced by lab-based XRD (samples D–I).

The structural quality of both perovskite and reduced samples were determined using Cu Kα_1_ XRD measurements performed on a PANalytical Empyrean X-ray diffractometer. Electrical transport measurements were performed using both a custom-built liquid-helium-cooled four-point probe measurement station (base temperature, 4 K) and a Quantum Design physical property measurement system (base temperature, 2 K). Finite-size factors were accounted for using the methods discussed elsewhere^48^. Contacts were prepared by either application of a dot of indium metal underneath a gold contact pin or by ultrasonic aluminium wire bonding.

### RSXS measurements

The RSXS measurements were performed at the REIXS beamline of the Canadian Light Source on a four-circle diffractometer in an ultrahigh-vacuum chamber (*P* < 5 × 10^−10^ torr). The nominal photon flux and energy resolution were *I*_0_ = 5 × 10^11^ photons s^–1^ and Δ*E*/*E* ≈ 2 × 10^−4^, respectively. The incoming X-ray polarization was selected to be in either the *σ* (*ε*_⊥_ scattering plane) or π (*ε*_∥_ scattering plane) configuration with the polarization of scattered X-rays unmeasured. The measurements were conducted at photon energies between 815 and 1,050 eV with either a microchannel plate or a silicon drift detector with an angular acceptance of 0.9°. The silicon drift detector is an energy-resolved detector, with a resolution larger than 50 eV, which allows for the removal of the substantial oxygen fluorescence background produced by the SrTiO_3_ substrate. A precise alignment to the film crystallographic axes was achieved by detecting the (001), (101) and $(\overline{1}01)$ Bragg peaks of the SrTiO_3_ substrate with an energy of 2.5 keV.

Additional RSXS measurements (Supplementary Information) were performed at beamline 8.0.1 of the Advanced Light Source. For these measurements, a fixed π-polarization geometry was used and the nominal photon flux and energy resolution were ~10^13^ photons s^–1^ and Δ*E*/*E* ≈ 7 × 10^−4^, respectively. The fluorescence yield and scattering signal were recorded using a GaAsP photodiode (with an aluminium window to block visible light and photoelectrons) mounted at 100 mm from the sample with an acceptance angle of 3°. To consistently calibrate the photon energies between different beamlines, a perovskite–nickelate sample (sample A) was measured at both endstations as an energy reference. The energy scale was then defined with respect to the NdNiO_3_ Ni L_3_ peaks at 852.6 and 854.3 eV and O K prepeak at 527.9 eV to align with prior literature measurements^9,10,28,49^. All the momenta are quoted in r.l.u. relative to the NdNiO_2_ lattice, where *a* = 3.905 Å and *c* = 3.286 Å.

All the RSXS traces in the manuscript, with the exception of the data shown in Fig. 2a, are presented as unsubtracted raw data, namely, *I*/*I*_0_, where *I* is the intensity measured on the detector and *I*_0_ is the incident-beam intensity determined from the current on a mesh located upstream on the beamline. In Fig. 2a, a cubic background was subtracted only from sample D, to better compare it with sample C. In Fig. 3d, the temperature dependence of the peak areas and correlation lengths were obtained after subtracting a fixed, temperature-independent second- or third-order polynomial background, which was unchanged (apart from a scaling factor) for each sample. Supplementary Fig. 4 shows the raw data for these temperature series.

### Spatially resolved hard XRD measurements

Scanning X-ray microdiffraction measurements were conducted at beamline 26-ID of the Advanced Photon Source at Argonne National Laboratory. A liquid-nitrogen-cooled Si(111) double-crystal monochromator was used to achieve an energy resolution of Δ*E*/*E* = 10^−4^ at a photon energy of 10.0 keV, and the scattered X-ray signal was recorded with an area detector, each pixel extending 0.00075 Å^−1^ along the Ewald sphere. The collimated X-ray beam was reduced to a spot size of 100 μm (full-width at half-maximum) using slits, yielding a flux of ~10^10^ photons s^–1^. The beam was vertically rastered along the sample, perpendicular to the scattering plane, to minimize the beam footprint and ensure that the spatial resolution of the linescan matched the beamwidth. A three-dimensional reciprocal-space map was obtained every 100 μm along a 4 mm line by rocking the sample by 4° per step in 41 steps and measuring a spatial linescan at each angle; Supplementary Fig. 11b shows projections of the map onto the L axis of the substrate/film.

### STEM measurements

STEM characterization was performed on a cross-sectional lamella prepared with the standard focused-ion-beam lift-out procedure using a Thermo Fisher Helios G4 UX focused ion beam. High-angle annular dark-field imaging and annular bright-field–STEM imaging were performed on a Cs-corrected Thermo Fisher Scientific Spectra instrument at 300 kV and 30 mrad probe convergence semiangle. For high-precision structural measurements, a series of 40 rapid-frame images were acquired and subsequently realigned and averaged by a method of rigid registration optimized to prevent lattice hops^50^, resulting in a high-signal-to-noise-ratio, high-fidelity image of the atomic lattice.

## Online content

Any methods, additional references, Nature Portfolio reporting summaries, source data, extended data, supplementary information, acknowledgements, peer review information; details of author contributions and competing interests; and statements of data and code availability are available at 10.1038/s41563-024-01797-0.

### Supplementary information

Supplementary Information Supplementary Figs. 1–13 and discussion.

### Source data

Source Data Fig. 1 Source data for Fig. 1.

Source Data Fig. 2 Source data for Fig. 2.

Source Data Fig. 3 Source data for Fig. 3.

Source Data Fig. 4 Source data (TIFF files) for Fig. 4.

## Data Availability

All data needed to evaluate the conclusions of this study are available in this Article and its Supplementary Information. Datasets generated and analysed during the course of this study are available at 10.34863/r44d-xt74. Source data are provided with this paper.
