# Publisher Correction: Absence of 3*a*_0_ charge density wave order in the infinite-layer nickelate NdNiO_2_

**DOI:** 10.1038/s41563-024-01832-0

**Published:** 2024-02-12

**Authors:** C. T. Parzyck, N. K. Gupta, Y. Wu, V. Anil, L. Bhatt, M. Bouliane, R. Gong, B. Z. Gregory, A. Luo, R. Sutarto, F. He, Y.-D. Chuang, T. Zhou, G. Herranz, L. F. Kourkoutis, A. Singer, D. G. Schlom, D. G. Hawthorn, K. M. Shen

**Affiliations:** 1 Laboratory of Atomic and Solid State Physics, Department of Physics, Cornell University, Ithaca, NY USA; 2 Department of Physics and Astronomy, University of Waterloo, Waterloo, Ontario Canada; 3 School of Applied and Engineering Physics, Cornell University, Ithaca, NY USA; 4 Department of Materials Science and Engineering, Cornell University, Ithaca, NY USA; 5 Canadian Light Source, Saskatoon, Saskatchewan Canada; 6 Advanced Light Source, Lawrence Berkeley National Laboratory, Berkeley, CA USA; 7 Center for Nanoscale Materials, Argonne National Laboratory, Lemont, IL USA; 8 Institut de Ciència de Materials de Barcelona (ICMAB-CSIC), Bellaterra, Spain; 9 Kavli Institute at Cornell for Nanoscale Science, Cornell University, Ithaca, NY USA; 10 Leibniz-Institut für Kristallzüchtung, Berlin, Germany

**Keywords:** Superconducting properties and materials, Electronic properties and materials

Correction to: *Nature Materials* 10.1038/s41563-024-01797-0, published online 26 January 2024.

In the version of the article initially published, in the top panel of Fig. 3a, “(0.31, 0, 0.30)” previously read “(–0.31, 0, 0.30)”. This has now been corrected in the HTML and PDF versions of the article.

