# Supplementary material for: Absence of 3a0 charge density wave order in the infinite-layer nickelate NdNiO2
Source: Nat Mater. 2024 Jan 26;23(4):486–91. doi: 10.1038/s41563-024-01797-0 (PMC10990928; doi:10.1038/s41563-024-01797-0)
Supplement: Supplementary file 1 — Supplementary Figs. 1–13 and discussion. [file 41563_2024_1797_MOESM1_ESM.pdf]

nature materials

Article https://doi.org/10.1038/s41563-024-01797-0

Absence of $3a_0$ charge density wave order in the infinite-layer nickelate $NdNiO_2$

In the format provided by the authors and unedited

CONTENTS

I. XAS Analysis and Intermediate Phase Structures 2

II. Additional data and measurements on samples A-F 5
A. Temperature Dependence 5
B. Out-of-plane Momentum Transfer 5
C. Energy Dependence of the Resonant Feature 6
D. Additional Null Results 7

III. Characterization and measurement of samples G-J 8
A. Null Results on Additional Samples 8
B. Position Dependent RSXS and HXRD Measurements 9
C. Characterization of Sample J 10

References 11

I. XAS ANALYSIS AND INTERMEDIATE PHASE STRUCTURES

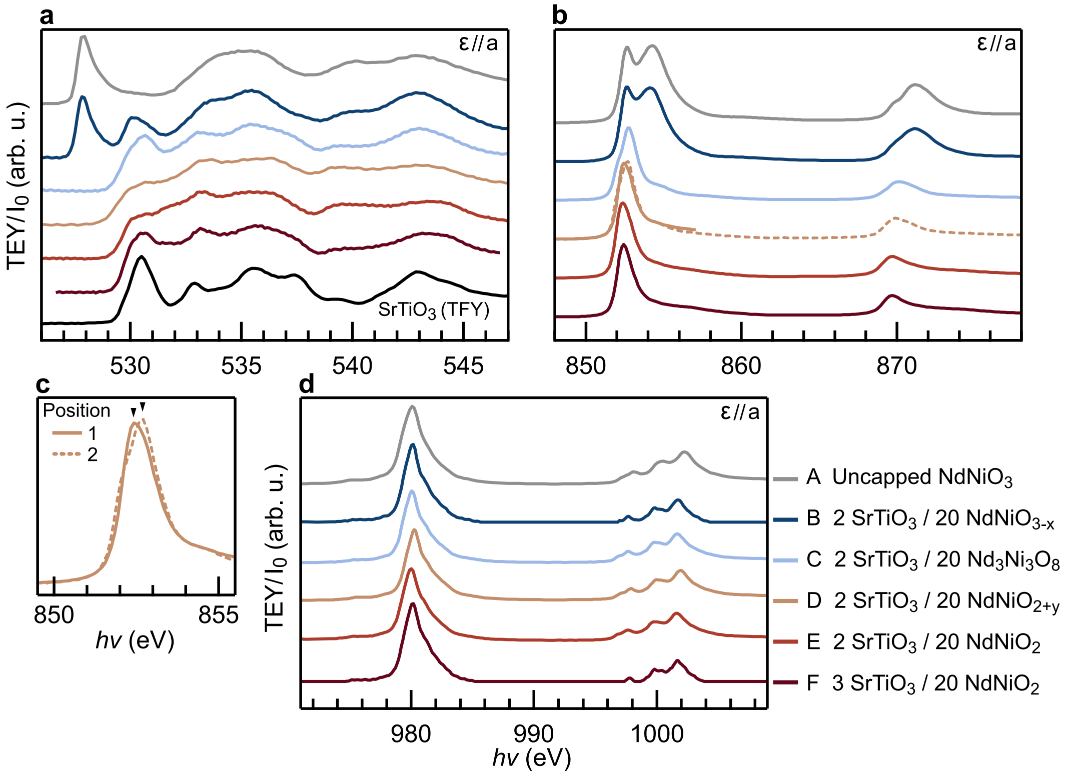

FIG. S1. Additional XAS measurements taken with the electric field, ε, parallel to the sample a-axis. (a) Total electron yield (TEY) Measurements at the oxygen K-edge for all samples presented in the main text. Total fluorescence yield (TFY) spectrum of a bare $SrTiO_3$ substrate is provided for reference. (b) Extended range spectrum at the nickel L_2 and L_3 edges. (c) Zoom in on Ni L_3 XAS for sample D, showing spectra obtained at two positions near the sample center. (d) TEY spectra at the neodymium M_4 and M_5 edges. All spectra were collected at normal incidence at temperatures of either 20 K or 78 K; traces have been rescaled and offset for clarity.

Here we present additional x-ray absorption spectroscopy (XAS) measurements on the samples from the main text. Larger range XAS measurements at the oxygen K, nickel L, and neodymium M edges are shown in Fig. S1. All spectra were collected at normal incidence with either a silicon drift detector or photodiode at either 20 or 78 K. Measurements were performed at both beamline 8.0.1 of the Advanced Light Source and the REIXS beamline of the Canadian Light Source and a common $NdNiO_3$ samples (Sample A) was measured at both beamlines as an energy reference. At the O K edge, samples A and B show a strong pre-peak associated with the O $2p$ - Ni $3d$ hybridization [1, 2] typical of the the perovskite nickelates. Conversely, samples C-F show no evidence of a prepeak feature at $\sim$ 528 eV, indicative of full conversion of the original perovskite to other phases [3]. At the Ni L edge, a split-peak structure at the nickel L_3 edge (851-855 eV) is observed in both samples A and B, as is expected for the perovskite nickelate, and no additional peaks associated with the formation of NiO are observable, indicating complete oxidation of the as-grown perovskite samples. The infinite-layer samples (D-F) show the typical single peak structure reported in the literature [4, 5]. Two spectra from different positions near the sample center are reported for Sample D in Fig. S1c. The two positions show slightly different XAS profiles at the L_3 edge with a peaks at 852.4 eV and 852.7 eV at positions 1 and 2, respectively. The resonant feature at $\mathbf{q} = (1/3, 0)$ was observed at both positions on the sample with similar correlation lengths ($\xi_2 \sim \xi_1$) but different amplitudes ($A_2 \sim 3A_1$). Finally, the neodymium M edges, shown in Fig. S1d, show little variation across the sample series.

In addition to the TEY measurements presented in Fig. S1, which were performed on all samples, partial fluorescence yield (PFY) measurements at the Ni L edge were also measured on samples B, C and F using a silicon drift detector [6, 7]. Selecting fluorescence from Ni L edge emission, and eliminating emission from oxygen or titanium, enables measurement of emission from the sample without contributions from the $SrTiO_3$ (STO) substrate or capping layer. These measurements were performed with both σ and π polarized light for a range of incident angles spanning grazing incidence ($\theta = 10°$) to normal incidence ($\theta = 90°$), such that π polarized light at grazing incidence probes with the

electric field approximately parallel to the c-axis of the sample.

For a thin film of thickness d_{NNO}, the intensity of the Ni L edge partial fluorescence yield is given by

$$\begin{aligned} I_{NiL,PFY} &\propto \frac{\mu_{NiL,i}}{\sin\alpha}\left(\int_0^{d_{NNO}} e^{-\left(\frac{\mu_{tot,i}}{\sin\alpha}+\frac{\mu_{tot,f}}{\sin\beta}\right)z}dz\right) \\ &= \frac{\mu_{NiL,i}}{\sin\alpha}\left(\frac{1}{\frac{\mu_{tot,i}}{\sin\alpha}+\frac{\mu_{tot,f}}{\sin\beta}}\right)\left(1-e^{-\left(\frac{\mu_{tot,i}}{\sin\alpha}+\frac{\mu_{tot,f}}{\sin\beta}\right)d_{NNO}}\right) \\ &= \frac{\mu_{NiL,i}d_{NNO}}{\sin\alpha}\left(1-\frac{1}{2}\left(\frac{\mu_{tot,i}}{\sin\alpha}+\frac{\mu_{tot,f}}{\sin\beta}\right)d_{NNO}+\frac{1}{3}\left(\frac{\mu_{tot,i}}{\sin\alpha}+\frac{\mu_{tot,f}}{\sin\beta}\right)^2 d_{NNO}^2+...\right), \end{aligned} \quad (1)$$

where $\mu_{tot,i}(\mu_{tot,f})$ is the total linear x-ray absorption co-efficient of the NNO_x layer at the incident(emitted) photon energy and $\mu_{NiL,i}$ is the contribution to the Ni L absorption coefficient of $NdNiO_x$ layer from excitation of $2p$ core electrons into unoccupied states. In our samples, the thickness of the STO capping layer $d_{STO} \sim 7-12$Å is thin relative to $1/\mu_{STO} \sim 2800$Å. Accordingly, the STO layer provides negligible absorption of the incident and scattered beam. As shown in equation 1, the intensity of the Ni L edge PFY will be proportional to $\mu_{NiL,i}$, but will be subject to a self-absorption correction depending on the values of $\mu_{tot,i}$, $\mu_{tot,f}$, α, β and d_{NNO}.

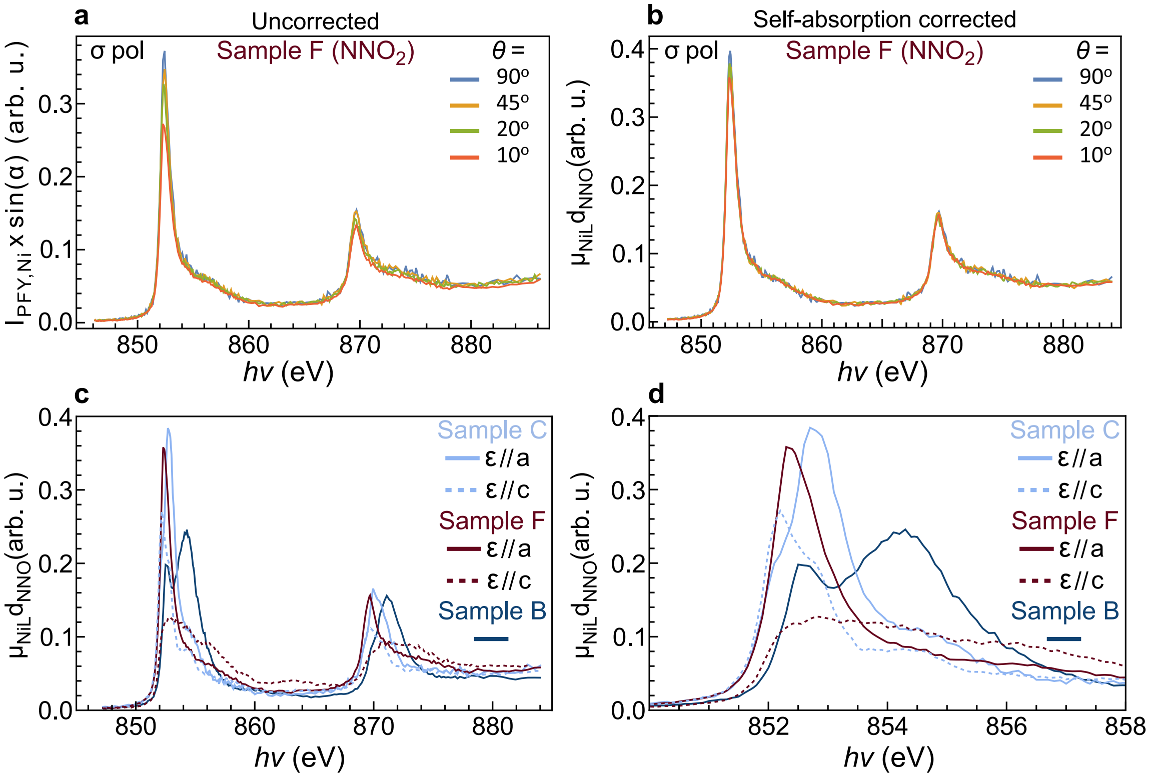

FIG. S2. Ni L edge partial fluorescence yield measurements of the x-ray absorption. (a) The Ni L edge partial fluorescence of sample F as a function of incidence angle, α with σ polarized light (with the electric field, ε parallel to a). Measurements are shown normalized to the incident beam intensity, I_0 and $\sin\alpha$. (b) $\mu_{NiL,i}$ determined from the data in (a) by applying a self-absorption correction. (c) and (d) $\mu_{NiL,i}$ as a function of energy for sample C and F for both $\varepsilon \parallel a$ and $\varepsilon \parallel c$. Data for $\varepsilon \parallel c$ were deduced from measurements at grazing incidence ($\alpha = 10°$) with π polarized light.

In Fig. S2, the raw PFY data for sample F are shown as a function of angle of incidence, α, for σ incident light. Here the data are normalized only to $\sin\alpha$ and the incident beam intensity, I_0. The good agreement between measurements at different α values above the L_3 edge indicates that self absorption corrections are not too large ($> 10\%$). At the peak of the L_3 edge at grazing incidence, self absorption can reduce the peak intensity by $\sim 25\%$, for our 20 u.c. thick samples. This self absorption effect can be partially corrected by scaling the PFY data to tabulated values of the absorption co-efficient above the Ni L edge [8] and inverting equation 1. This procedure gives $\mu_{NiL,i}d_{NNO}$ shown

in Fig. S2b, showing good agreement between measurements at different values of α. Applying this procedure to sample B, C and F using σ and π polarized light at grazing incidence provides a good comparison of the energy and polarization dependence of the XAS between samples, notably without any free parameters or the need to otherwise scale or offset measurements taken on different samples. As shown in Fig. S2c,d, the $NdNiO_2$ sample (Sample F) shows dichroism consistent with literature, with a large peak for $E \parallel a$ at 851.2 eV that is diminished with $E \parallel c$, consistent with Ni^{1+} and holes in $d_{x^2-y^2}$ orbitals.

In contrast, sample C exhibits a peak at slightly higher energy (852.8 eV) than sample F for $E \parallel a$. Moreover, with $E \parallel c$, sample C exhibits a peak at lower energy (852.2 eV) than the peaks in both samples C and F with $E \parallel a$. Moreover, the energy dependence and dichroism, contrasts with that of perovskite Ni^{3+} (sample B). This is suggestive of at least some concentration of Ni^{2+} sites in sample C,[9] with holes in both $d_{x^2-y^2}$ and $d_{3z^2-r^2}$ orbitals. Unlike Ni^{2+} in NiO, dichroism indicates a crystal field with symmetry lower than O_h, such as Ni residing in pyramidal sites, with $d_{3z^2-r^2}$ orbitals having a lower energy than the $d_{x^2-y^2}$ states.

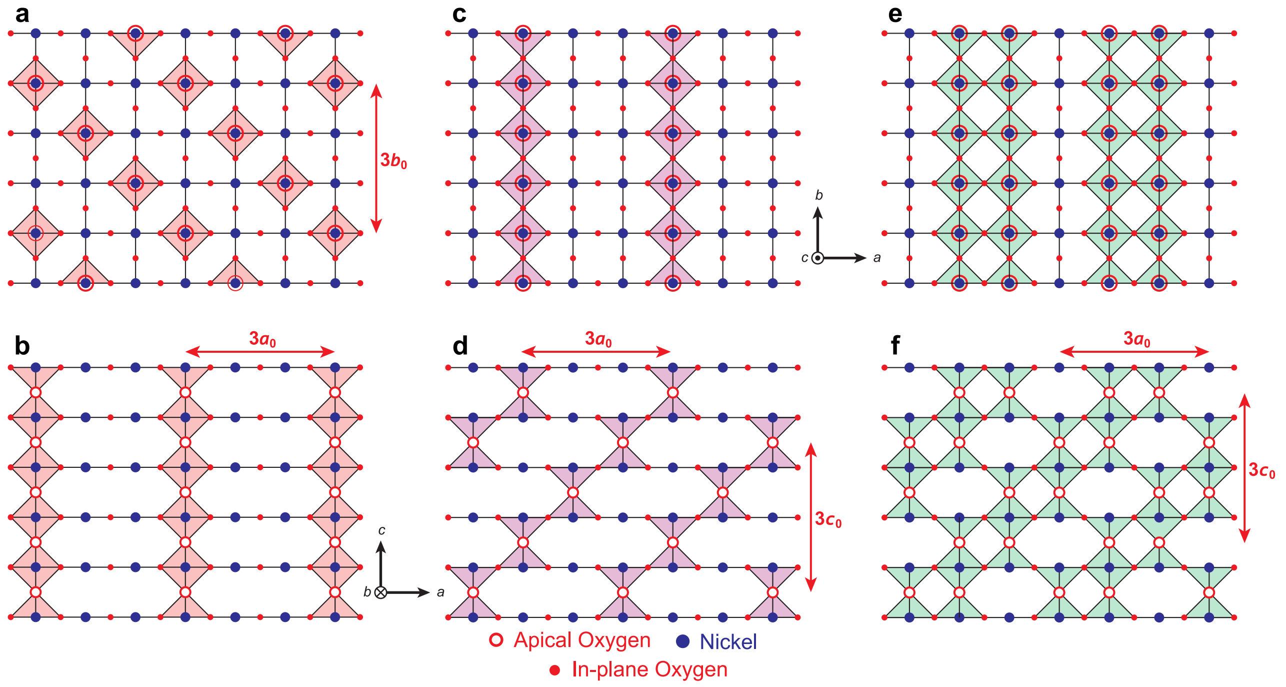

FIG. S3. Proposed structures for they oxygen ordering in $NdNiO_{2+x}$. (a-b) The proposed structure of $Nd_3Ni_3O_7$, from Moriga et al. based on x-ray powder refinement, with octahedral coordination of the nickel ions. (c-d) A proposed structure for $Nd_3Ni_3O_7$ consisting of alternating square-planar and pyrimidal coordination of the nickel ions. (e-f) A proposed structure for $Nd_3Ni_3O_8$ consisting of alternating pyrimidal and octahedral coordination of the nickel ions.

In Fig. S3 we display three proposed structures for the oxygen ordering in intermediate phase nickelates. Fig. S3a,b, reproduces the structure for $(Nd,Pr)_3Ni_3O_7$ proposed based on bulk powder x-ray refinement in Ref. [10] with the remaining oxygen atoms forming octahedral chains along one axis. XAS measurements, detailed in Fig. S2, on sample C suggest a lower symmetry configuration of the nickel ions than the O_h configuration proposed by Moriga et al. [10]. As such we propose an alternate structure for $Nd_3Ni_3O_7$ / $SrTiO_3$ with alternating pyramidal and planar coordination of the nickel ions, and chains of missing apical oxygen ions running in the basal plane. This structure, depicted in panel (c-d), can be naturally extended via the addition of one additional oxygen ion per formula unit to a structure of $Nd_3Ni_3O_8$ which is consistent with the STEM results on Sample J presented in the main text, as shown in Fig. S3e,f. Prior studies of oxygen deficient brownmillerite-like structures, $(La,Sr)_2Co_2O_5$, indicate that the ordering of oxygen vacancies is sensitive to sample orientation and strain [11]. Furthermore, bulk electron diffraction studies of the reduction products of $LaNiO_3$ indicate the presence of several orderings, even in the same sample, including $La_2Ni_2O_5$, $La_4Ni_4O_{11}$, and $La_3Ni_3O_8$ [12, 13]. As such, it is plausible that any or all of these structures may occur in a given thin film sample, with their presence dictated by details of the sample (e.g. the presence or absence of defects in the perovskite precursor) or the reduction process itself (reducing agent, time, temperature). Detailed macro- and micro-structural analysis of the factors contributing to oxygen ordering in the infinite-layer nickelates, and topotactically reduced oxides generally, will likely play an important role in both stabilizing and understanding novel reduced oxides.

II. ADDITIONAL DATA AND MEASUREMENTS ON SAMPLES A-F

A. Temperature Dependence

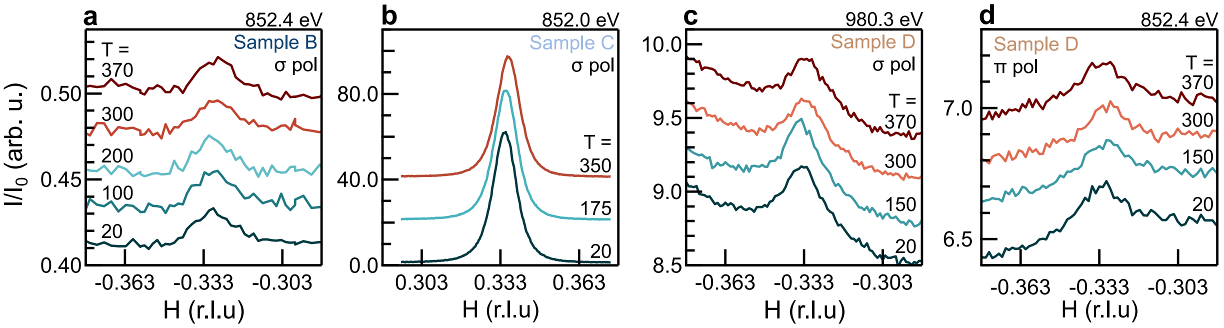

FIG. S4. Additional temperature dependent measurements of the resonant feature for samples B, C, and D of the main text. (a) Measurements of the lightly reduced perovskite sample, B, at the Ni L_3 edge. (b) Measurements of the intermediate oxygen ordered phase sample, C, at the Ni L_3 edge. (c-d) Measurements of the predominately infinite-layer sample, D, at the Nd M_5 and Ni L_3 edges, respectively. Data are presented without any background subtraction but traces have been vertically offset for clarity.

A selected set of the raw data from the temperature-dependent measurements depicted Fig. 3 of the main text are presented in Fig. S4. As can be seen in the raw data, there is little to no observable variation in the peak characteristics in any of samples B, C, or D as a function of temperature, which is consistent with the fitting results presented in the main text – only a weak dependence in the intensity and no observable trend in the correlation length. We note that the fitted correlation correlation lengths, $\xi = 2\pi/\Delta q$, observed here are somewhat larger then those reported previously by a factor of 3-5 [14–17]. Finally, we observe that in each temperature series that there is a slight shift in the peak position to higher q as the samples are warmed from 20 to 370 K. In sample C the shift is measured to be 0.0012 r.l.u. (0.3%) relative to $SrTiO_3$ in-plane lattice parameter. This shift is consistent with thermal expansion of the $SrTiO_3$ substrate as measured in Ref. [18]. The increase in the pseudocubic lattice parameter from ~ 3.899 Å at 20 K to 3.905 Å at 300 K shifts reciprocal lattice to higher q by $\sim 0.2\%$, in good agreeance with our observations.

B. Out-of-plane Momentum Transfer

Dependence on the out-of-plane momentum transfer, L, for samples B, C, and D is reported in Fig. S5; in all cases L is reported relative to the infinite-layer out-of-plane lattice parameter, $c = 3.286$ Å. In sample B, little change is observed as a function of of L over the range measured, indicating short correlation lengths out of the plane of the ordering. In contrast, in the film composed predominately of the ordered oxygen vacancy phase shows a relatively sharp L dependence, though with a larger correlation length ($\xi_{(0,0,L)}$ =4-6 nm) than in-plane ($\xi_{(H,0,0)} = 37$ nm). This is expected given the out-of-plane coherence is limited by the film thickness of ~ 7 nm. At 852.1 eV, the curve is peaked around L = 0.30 r.l.u., which gives a plane spacing of $d/3 = 3.65$ Å which is in agreement with the film c-axis lattice constant, $c = 3.659$ Å, measured by XRD (Fig. 1 of the main text). Note, the data shown in Fig. S5b are corrected for angle-dependent absorption of the incident and scattered beam by dividing the scattered intensity by

$$\frac{1 - e^{-\mu(1/\sin\alpha + 1/\sin\beta)d_{NNO}}}{\mu\left(1 + \frac{\sin\alpha}{\sin\beta}\right)}, \tag{2}$$

where α and β are the angles of incidence and emission, μ is the linear absorption coefficient and d_{NNO} is the thickness of the $NdNiO_x$ layer. This is done because the absorption can vary strongly with L for L scans, impacting the peak position in L. The L dependence of Sample D is, similar to sample B, not pronounced and changes in the peak intensity are comparable to that of the background shape; however, the intensity appears to be at a maximum between 0.280 and 0.333 r.l.u., at roughly $\sim 3.1(2)$ r.l.u which is qualitatively similar to prior measurements, showing a weak peak around $L \sim 1/3$.

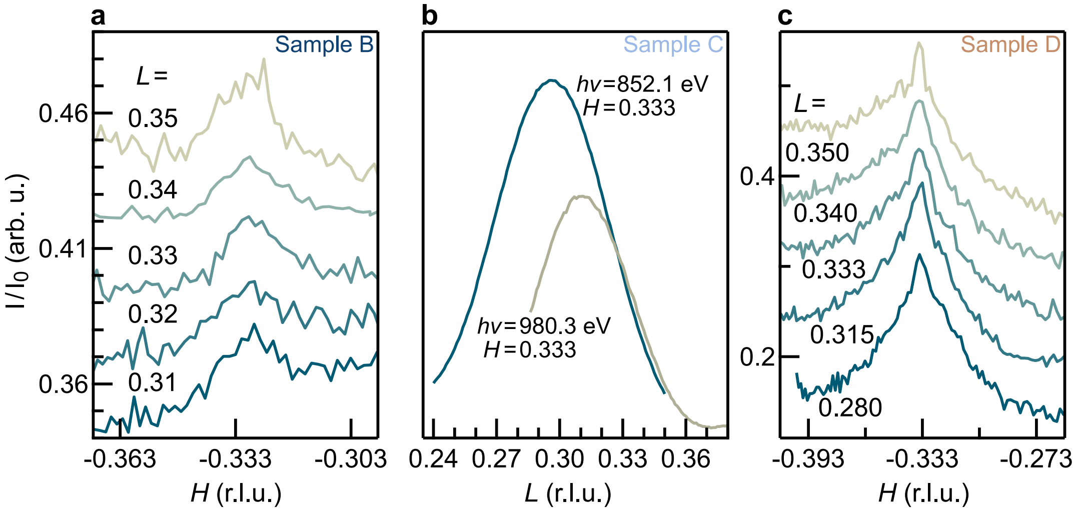

FIG. S5. L dependence of the resonant feature. (a) Rocking curves through (-0.333, 0, L) for different values of L on sample B from the main text. L scans at constant H for sample C at the Nd/Ni resonances, after application of an angle dependent absorption correction. (c) Rocking curves through $(-0.333, 0, L)$ at different L from sample D. Data are presented without any background subtraction but traces have been vertically offset for clarity.

C. Energy Dependence of the Resonant Feature

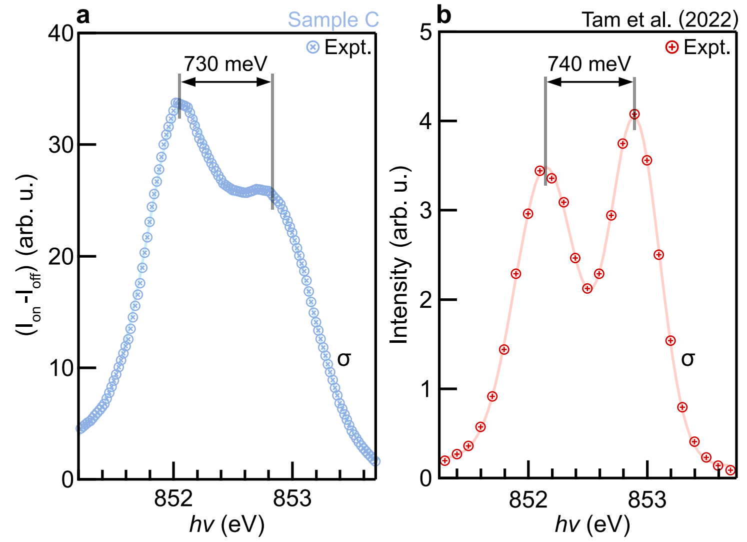

FIG. S6. Resonance profile comparison at the Ni L_3 edge. (a) Difference between the intensity at the scattering wavevector of $q = (0.333, 0, 0.290)$, $I_{\rm on}$, and off the peak at $q = (0.310, 0, 0.300)$, $I_{\rm off}$. (b) Data extracted and reproduced from Fig. 1e of Tam et al. (2022) [15]. Fitted peak positions are marked.

Additional data and a analysis of the resonant profiles of samples C and B are provided in Figs. S6 and S7. The resonance profile of the scattering peak (difference between the signal measured on the peak, and off – to remove contributions from the background fluorescence) for (intermediate phase) Sample C is depicted in Fig. S6a. The two peak structure, with a splitting of 730 meV, matches well with prior measurements of the resonance profile by resonant inelastic x-ray scattering in Tam et al. [15]. The data from this reference are reproduced in Fig. S6b, where the similar peak splitting of 740 meV is apparent. The similarity of these resonant profiles, including both the bifurcation and the overall energy width of the feature, suggests a common origin. The resonance profile, at fixed $\mathbf{q}$, of Sample C at the neodymium M edge is pictured in Fig. S7 and shows strong enhancement of the scattering intensity at both the M_4 and M_5 peaks. For reference, the fixed $\mathbf{q}$ resonance profile for the lightly reduced Sample B is shown in Fig.

S7b,c, where a small, but measurable, enhancement above the fluorescence is observable. Finally, rocking curves at a variety of photon energies well off of the Ni and Nd resonances for Sample B are provided in Fig. S7d. Just like samples C and D from the main text, the $\mathbf{q} = (1/3, 0)$ feature is weak, but observable well off resonance indicating a structural origin of this peak as well.

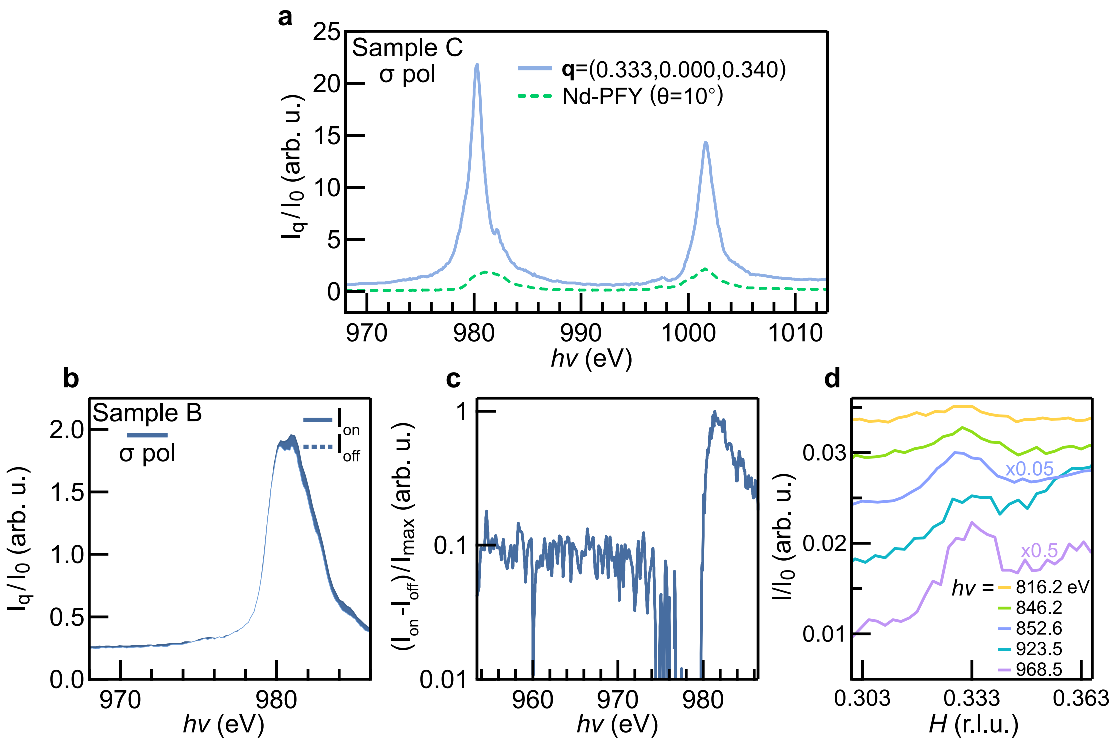

FIG. S7. Resonance profile comparison at the Nd M_5 edge. (a) Scattered intensity at fixed $\mathbf{q} = (0.333, 0.000, 0.340)$ as a function of photon energy, for sample C. Grazing incidence ($\theta = 10°$, σ polarization) neodymium PFY measurement is included as an intensity reference. (b) Resonant energy profile of sample B at the Nd M edge on the peak scattering wavevector, $I_{\rm on}$, and off, $I_{\rm off}$ (as a measure of the fluorescence background). (c) Difference between the intensity on and off the scattering wave vector, normalized to the maximum value. (d) Rocking curves on sample B, through $\mathbf{q} = (0.333, 0.000, 0.340)$, performed at different off-resonant energies – presented without any background subtraction but traces have been vertically offset for clarity..

D. Additional Null Results

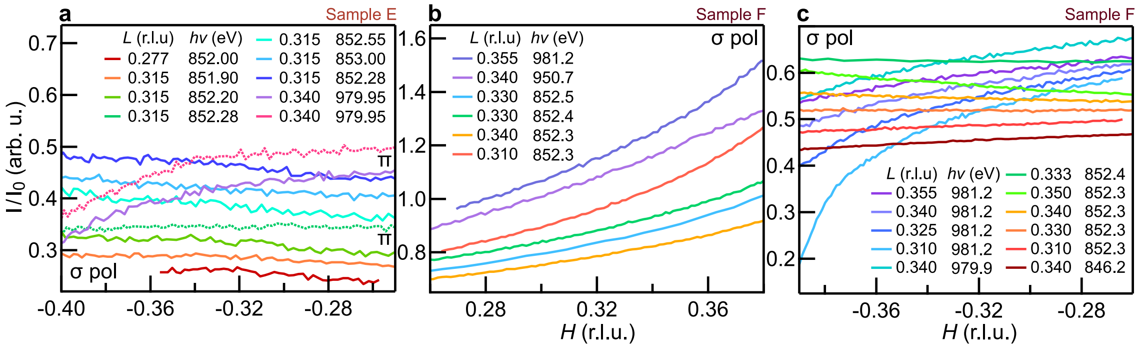

FIG. S8. Additional RSXS measurements performed on samples E and F. Rocking curves centered at $(\pm 1/3, 0, L)$ at multiple values of L and $h\nu$ show no indication of a peak at the nominal ordering wave-vector, $\mathbf{q} = (1/3, 0)$, in either sample. Traces are presented without any background subtraction or rescaling have been vertically offset for clarity.

Finally, in Fig. S8 we show additional null results on samples E and F from the main text. Following careful align-

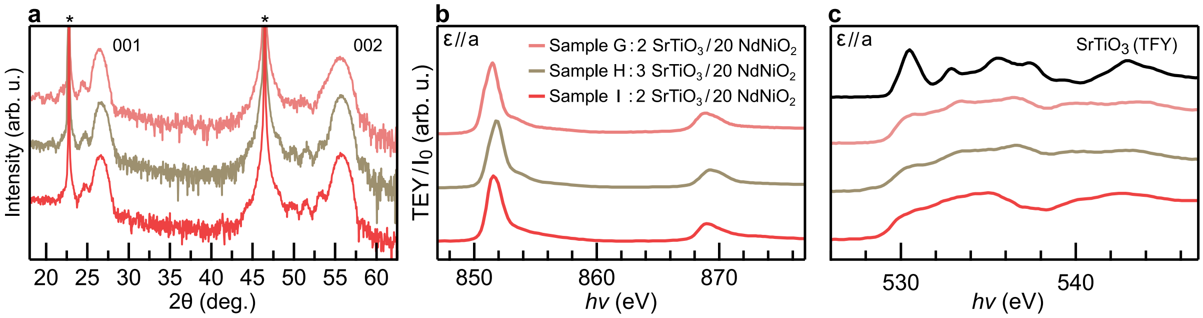

FIG. S9. Characterization of samples G, H, and I. (a) Lab based XRD measurements of all three samples showing the (001) and (002) diffraction peaks; $SrTiO_3$ substrate peaks are marked with a star (*). b) Nickel L edge XAS-TEY measurements showing the single peak structure of $NdNiO_2$. (c) Oxygen K edge XAS-TEY measurements showing no pre-peak at ~ 528 eV, a TFY measurement of a $SrTiO_3$ substrate is included for reference. All plotted spectra were taken with the electric field ε, parallel to the film a-axis. Traces have been vertically offset for clarity.

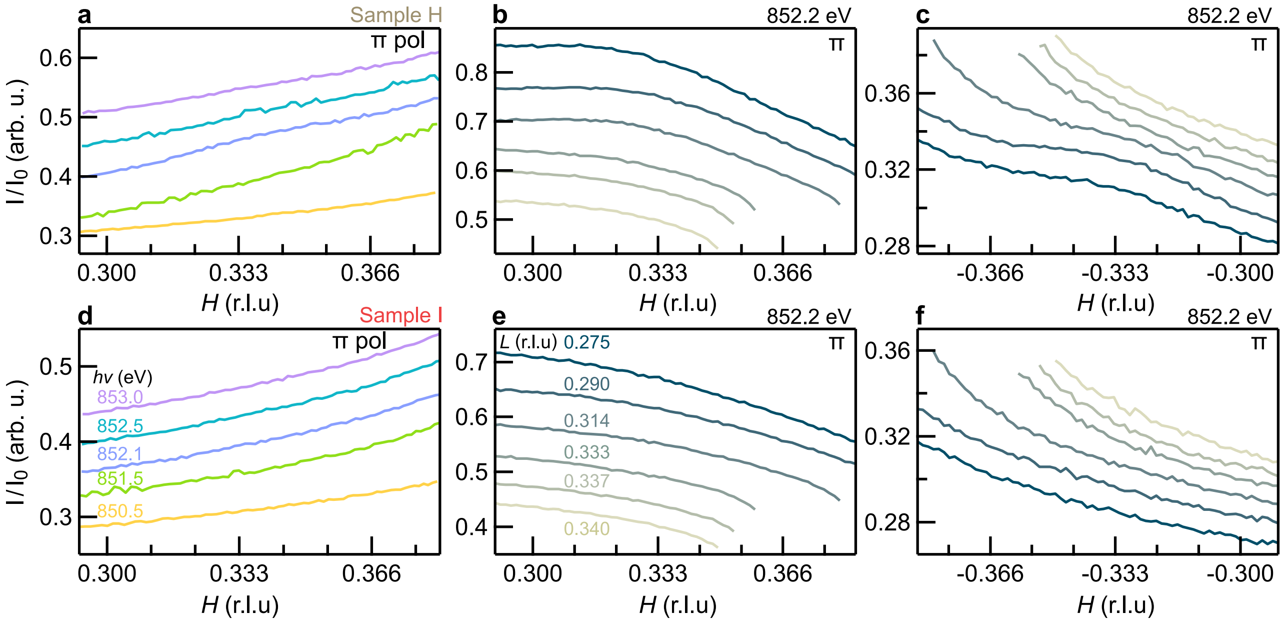

FIG. S10. Additional RSXS measurements performed on samples H and I at beamline 8.0.1 of the Advanced Light Source using a silicon photodiode detector. (a) Rocking curves through $\mathbf{q} = (0.333, 0, 0.315)$ at different photon energies. (b-c) H scans at constant L at different values of L ranging from 0.275 to 0.340 r.l.u about $H = +1/3$ and $H = -1/3$, respectively. (d-f) Same scans performed on sample I. Data are presented without any background subtraction but traces have been vertically offset for clarity.

ment to the substrate Bragg peaks a series of rocking curves was performed on either sample including measurements at and around both the Ni L_3 and Nd M_5 edges. No peaks were observed at any energy or at any of the L values tested in the vicinity of previous reports near $\mathbf{q} = (1/3, 0, 1/3)$.

III. CHARACTERIZATION AND MEASUREMENT OF SAMPLES G-J

A. Null Results on Additional Samples

In this section we describe measurements on additional samples not featured in the main text, Samples G-I, as well as characterization details for the sample whose TEM profile is included in Fig. 4 of the main text, Sample J. Cu K-α XRD measurements of samples G, H, and I are shown in Fig. S9a. Samples G, H, and I were all prepared in nominally the same way as samples D-F in the main text with exception that sample G was exposed to the atomic hydrogen

beam for a slightly lower time: 10 minutes, in contrast to the typical 12-15. As a result, while the sample appears predominately infinite-layer in composition, there are some weak shoulders visible decorating the (002) Bragg peak at $2\theta = 50.5^\circ$ and 53.5°, this is in contrast to samples H and I which show only the Pendellösung fringe pattern of the main text samples. XAS measurements at the Ni L and O K edges for the three samples are shown in Fig. S9b,c, and have the expected single Ni L_3 peak at $\sim$ 852 eV associated with $NdNiO_2$ and no visible O K edge pre-peak associated with unreduced $NdNiO_3$. Resonant scattering measurements on samples H and I are presented in Fig. S10, and spatially resolved soft and hard x-ray measurements on sample G are detailed in Fig. S11. Sample H, shows weak signatures of a resonant feature at $H = \pm 1/3$ which is only barely visible in the rocking curve at $h\nu = 852.1$ eV and not visible at most values of L except between 0.275 and 0.314 r.l.u., as can be seen in panels (b-c) – this peak has amplitude only 4% above the background and FWHM of > 0.04 r.l.u ($\xi < 10$ nm). Sample I, shown in Fig. S10d,e,f, shows no evidence of any resonant features at any values of $h\nu$ or L.

B. Position Dependent RSXS and HXRD Measurements

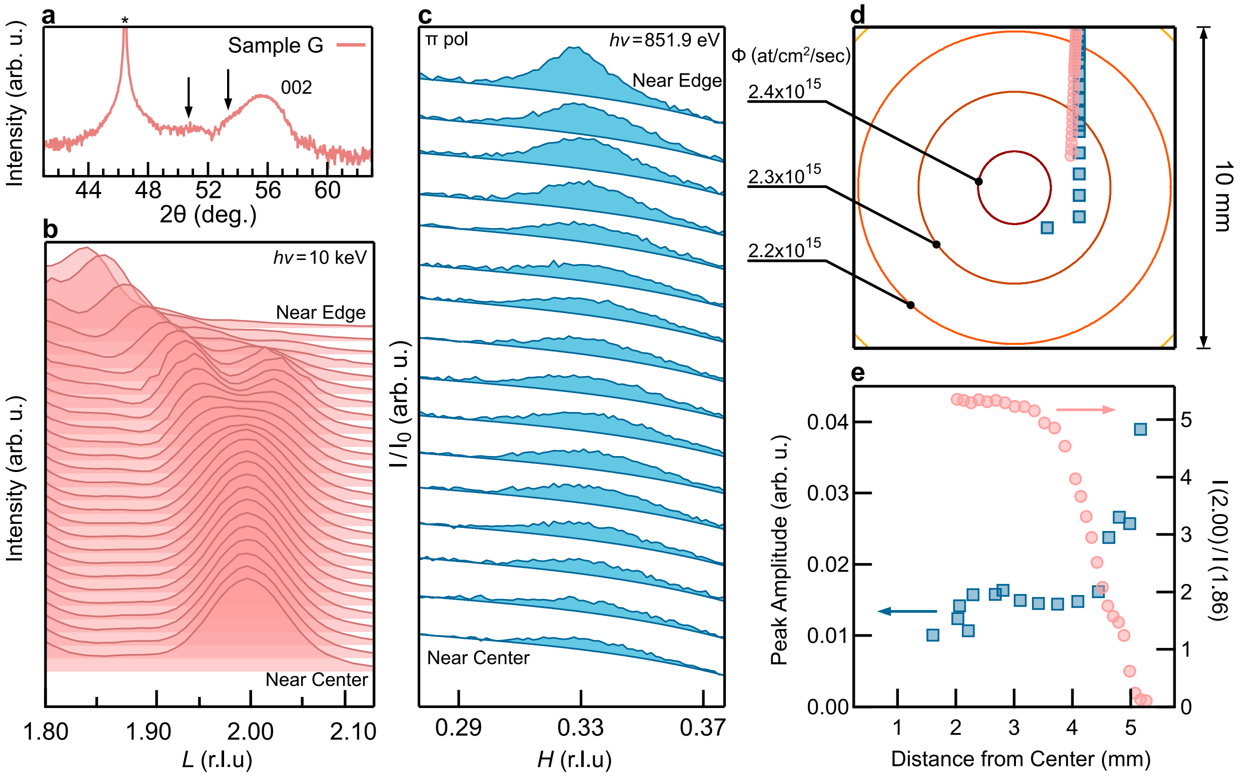

FIG. S11. Spatially resolved hard and soft x-ray measurements of Sample G. (a) Lab-based Cu K-α x-ray diffraction measurement with a beam width > 10 mm, covering the entire sample. The $SrTiO_3$ substrate peak is marked with a star (*), and positions of intermediate-phase peaks are indicated by arrows. (b) Sequence of hard x-ray diffraction (00L) scans taken at positions ranging from near the sample edge to near the sample center. (c) Position-dependent soft x-ray rocking curves through $\mathbf{q} = (0.333, 0, 0.315)$ at the Ni L_3 resonance. Traces are presented with a fit cubic background and have been offset for clarity. (d) Diagram of a 10×10 mm sample with the measurement positions from (b) and (c) marked. Estimated hydrogen fluxes at different positions on the sample are marked. (e) Resonant scattering peak amplitude and $NdNiO_2$ peak amplitude as a function of distance from the sample center.

Due to the expanding nature of the atomic hydrogen source, the flux at the sample surface is not perfectly uniform, and a drop of 15-20% across the sample is expected based on characterization data of the source [19, 20]. Additionally, as the sample is suspended at the corners, and heated radiatively from behind, there is typically a thermal gradient across the sample with the corners remaining slightly cooler then the center. Both of these factors (reduced flux and sample temperature) are expected to cause the edges of the sample to reduce more slowly then the sample center. To test this hypothesis, Sample G was reduced in a similar manner to the other $NdNiO_2$ samples reported in this study, but for a slightly shorter duration. As a result some impurity peaks remain visible in area averaged XRD

measurements of the (002) peak, indicated in Fig. S11a. In Fig. S11b micro-spot diffraction measurements about the (002) Bragg peak are reported where the x-ray beam (100 μm $\times$ 100 μm) is rastered from the sample edge towards the center. These spatially resolved measurements confirm that the sample edge has a clustering of intermediate-phase peaks with lattice constants greater than $NdNiO_2$, even though the sample center appears well reduced to the infinite-layer phase. Later, this same sample was remeasured at low photon energies corresponding to the Ni L_3 resonance. These spatially resolved RSXS measurements (spot size: vertical $\times$ horizontal = 30 μm $\times$ 200 μm), taken at positions coincident with the hard x-ray measurements, are detailed in panel C and the positions on the sample surface are indicated in D. As expected, the $\mathbf{q} = (1/3, 0)$ peak is strongest near the sample edge and tapers off approaching the center. These results are summarized in Fig. S11e where the fitted amplitude of the $(1/3, 0)$ peak is compared to the intensity of the (002) Bragg peak (normalized by the off-peak intensity). The anticorrelation between the presence of the $NdNiO_2$ phase and the resonant feature strongly indicates that this feature is not intrinsic to the infinite-layer phase.

C. Characterization of Sample J

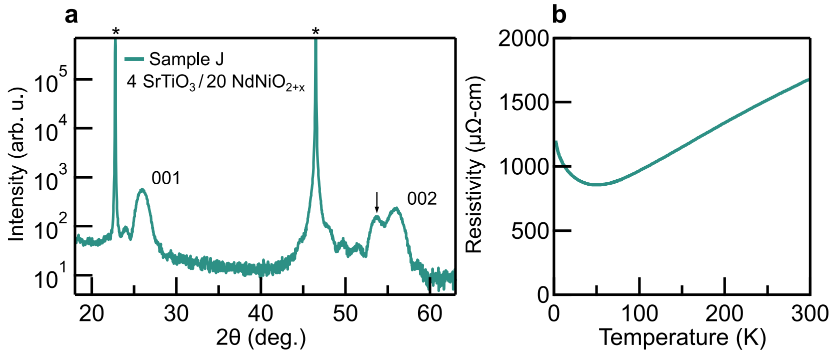

FIG. S12. Characterization of Sample J. (a) Lab-based Cu K-α X-ray diffraction measurements of the (001) and (002) reflections. $SrTiO_3$ substrate peaks are marked with a star (*), and the arrow indicates the peak position of the incompletely reduced phase. (b) Temperature dependent resistivity.

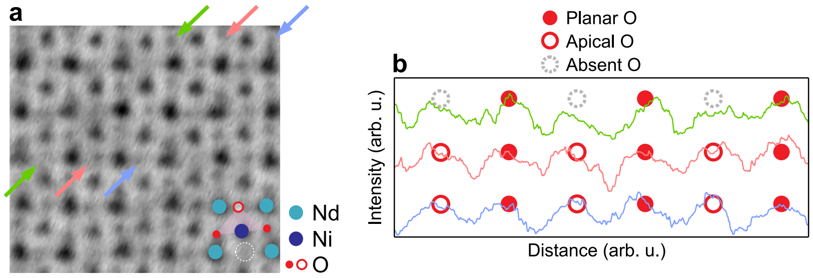

FIG. S13. 1D profiles of the ABF-TEM image of Sample J presented in Fig. 4(d) of the main text. (a) Reproduction fo the ABF image from the main text with diagonal paths marked (arrows). (b) Extracted line profiles along the marked paths with the expected positions of apical and planar oxygen ions marked.

Basic characterization of Sample J is provided in Fig. S12. Lab-based XRD measurements indicate that the sample is partially reduced, with a strong sharp peak occurring at the expected position of $NdNiO_2$, $2\theta = 55.86°$. An additional phase is clearly present in the film, as evidenced by the side peak at $2\theta = 53.67°$ (NB: This is nearly the same peak position of the shoulder observed in Sample G in Fig. S11a. The sample resistivity, shown in panel B is qualitatively similar to $NdNiO_2$ films presented here and in the literature, however the minimum resistivity, 860 $\mu\Omega$-cm, and low temperature resistivity, $\rho(2\text{ K}) = 1200\mu\Omega$-cm, are higher then observed in samples D, E, and F – which is consistent with inclusions of insulating intermediate phases in the film. The film was grown and reduced using the same conditions as D; however, it possesses a thicker, 4 u.c. $SrTiO_3$, capping layer – the presence of additional reduction products in the film is not unexpected, given that the conditions were not adjusted to account for the thicker capping layer. Fig. S13(b) shows a series of 1-D profiles along diagonals of the ABF-TEM image presented in

Fig. 4(d) of the main text. The alternating pattern of reduced intensity along the path marked in green indicates the missing apical oxygen ions in every third diagonal, consistent with the period three distortions in the Nd sublattice visible in the HAADF-TEM image and its fourier transform.

---

[1] F. M. F. de Groot, M. Grioni, J. C. Fuggle, J. Ghijsen, G. A. Sawatzky, and H. Petersen, Oxygen 1s x-ray-absorption edges of transition-metal oxides, Physical Review B **40**, 5715 (1989).

[2] N. Palina, L. Wang, S. Dash, X. Yu, M. B. Breese, J. Wang, and A. Rusydi, Investigation of the metal-insulator transition in $NdNiO_3$ films by site-selective X-ray absorption spectroscopy, Nanoscale **9**, 6094 (2017).

[3] B. H. Goodge, D. Li, K. Lee, M. Osada, B. Y. Wang, G. A. Sawatzky, H. Y. Hwang, and L. F. Kourkoutis, Doping evolution of the Mott–Hubbard landscape in infinite-layer nickelates, Proc. Natl. Acad. Sci. **118**, e2007683118 (2021).

[4] Z. Chen, M. Osada, D. Li, E. M. Been, S. D. Chen, M. Hashimoto, D. Lu, S. K. Mo, K. Lee, B. Y. Wang, F. Rodolakis, J. L. McChesney, C. Jia, B. Moritz, T. P. Devereaux, H. Y. Hwang, and Z. X. Shen, Electronic structure of superconducting nickelates probed by resonant photoemission spectroscopy, Matter **5**, 1806 (2022), 2106.03963.

[5] M. Rossi, H. Lu, A. Nag, D. Li, M. Osada, K. Lee, B. Y. Wang, S. Agrestini, M. Garcia-Fernandez, J. J. Kas, Y.-D. Chuang, Z. X. Shen, H. Y. Hwang, B. Moritz, K.-J. Zhou, T. P. Devereaux, and W. S. Lee, Orbital and spin character of doped carriers in infinite-layer nickelates, Physical Review B **104**, L220505 (2021).

[6] S. Eisebitt, T. Böske, J.-E. Rubensson, and W. Eberhardt, Determination of absorption coefficients for concentrated samples by fluorescence detection, Phys. Rev. B **47**, 14103 (1993).

[7] A. J. Achkar, T. Z. Regier, H. Wadati, Y.-J. Kim, H. Zhang, and D. G. Hawthorn, Bulk sensitive x-ray absorption spectroscopy free of self-absorption effects, Phys. Rev. B **83**, 081106 (2011).

[8] C. Chantler, Detailed tabulation of atomic form factors, photoelectric absorption and scattering cross section, and mass attenuation coefficients in the vicinity of absorption edges in the soft x-ray(z=30-36, z=60-89, e=0.1 kev-10 kev), addressing convergence issues of earlier work., J. Phys. Chem. Ref. Data **29**, 597 (2000).

[9] M. W. Haverkort, N. Hollmann, I. P. Krug, and A. Tanaka, Symmetry analysis of magneto-optical effects: The case of x-ray diffraction and x-ray absorption at the transition metal $L_{2,3}$ edge, Phys. Rev. B **82**, 094403 (2010).

[10] T. Moriga, O. Usaka, I. Nakabayashi, Y. Hirashima, T. Kohno, S. Kikkawa, and F. Kanamaru, Reduction of the perovskite-type $LnNiO_3$ (Ln=Pr,Nd) to $Ln_3Ni_3O_7$ with monovalent nickel ions, Solid State Ionics **74**, 211 (1994).

[11] J. Gazquez, S. Bose, M. Sharma, M. A. Torija, S. J. Pennycook, C. Leighton, and M. Varela, Lattice mismatch accommodation via oxygen vacancy ordering in epitaxial $La_{0.5}Sr_{0.5}CoO_{3-\delta}$ thin films, APL Mater. **1**, 0 (2013).

[12] J. M. González-Calbet, M. J. Sayagués, and M. Vallet-Regí, An electron diffraction study of new phases in the $LaNiO_{3-x}$ system, Solid State Ionics **32-33**, 721 (1989).

[13] M. Sayagués, M. Vallet-Regí, A. Caneiro, and J. González-Calbet, Microstructural Characterization of the $LaNiO_{3-y}$ System, Journal of Solid State Chemistry **110**, 295 (1994).

[14] G. Krieger, L. Martinelli, S. Zeng, L. E. Chow, K. Kummer, R. Arpaia, M. Moretti Sala, N. B. Brookes, A. Ariando, N. Viart, M. Salluzzo, G. Ghiringhelli, and D. Preziosi, Charge and Spin Order Dichotomy in NdNiO2 Driven by the Capping Layer, Physical Review Letters **129**, 027002 (2022).

[15] C. C. Tam, J. Choi, X. Ding, S. Agrestini, A. Nag, M. Wu, B. Huang, H. Luo, P. Gao, M. García-Fernández, L. Qiao, and K.-J. Zhou, Charge density waves in infinite-layer $NdNiO_2$ nickelates, Nature Materials **21**, 1116 (2022).

[16] M. Rossi, M. Osada, J. Choi, S. Agrestini, D. Jost, Y. Lee, H. Lu, B. Y. Wang, K. Lee, A. Nag, Y.-D. Chuang, C.-T. Kuo, S.-J. Lee, B. Moritz, T. P. Devereaux, Z.-X. Shen, J.-S. Lee, K.-J. Zhou, H. Y. Hwang, and W.-S. Lee, A broken translational symmetry state in an infinite-layer nickelate, Nature Physics **18**, 869 (2022).

[17] X. Ren, R. Sutarto, Q. Gao, Q. Wang, J. Li, Y. Wang, T. Xiang, J. Hu, F.-C. Zhang, J. Chang, R. Comin, X. J. Zhou, and Z. Zhu, Symmetry of charge order in infinite-layer nickelates (2023), arXiv:2303.02865 [cond-mat.supr-con].

[18] R. Loetzsch, A. Lübcke, I. Uschmann, E. Förster, V. Große, M. Thuerk, T. Koettig, F. Schmidl, and P. Seidel, The cubic to tetragonal phase transition in $SrTiO_3$ single crystals near its surface under internal and external strains, Applied Physics Letters **96**, 071901 (2010).

[19] K. G. Tschersich and V. von Bonin, Formation of an atomic hydrogen beam by a hot capillary, Journal of Applied Physics **84**, 4065 (1998).

[20] K. G. Tschersich, J. P. Fleischhauer, and H. Schuler, Design and characterization of a thermal hydrogen atom source, Journal of Applied Physics **104**, 034908 (2008).
