# Supplementary figures and images for: Absence of 3a0 charge density wave order in the infinite-layer nickelate NdNiO2
Source: Nat Mater. 2024 Jan 26;23(4):486–91. doi: 10.1038/s41563-024-01797-0 (PMC10990928; doi:10.1038/s41563-024-01797-0)

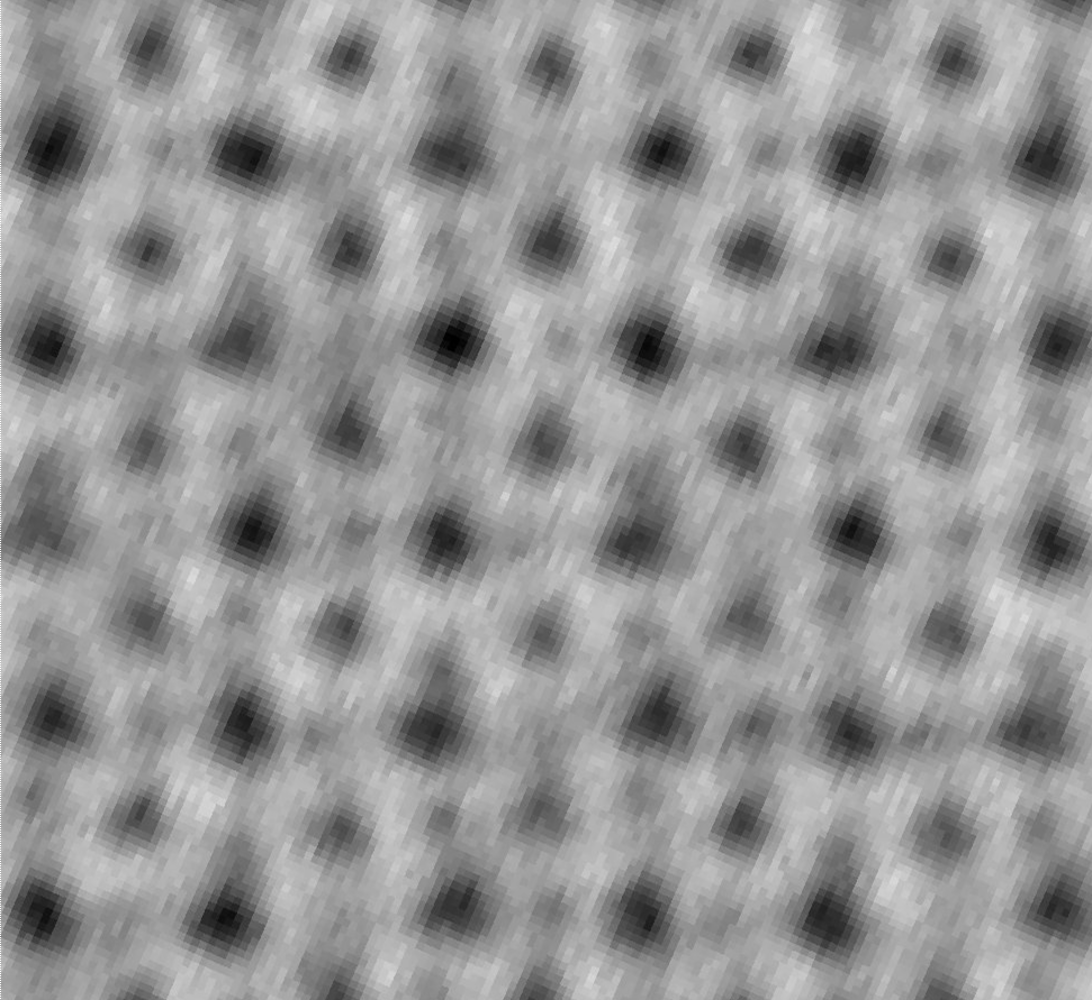

Supplement: Supplementary file 5 — Source data (TIFF files) for Fig. 4. [file 41563_2024_1797_MOESM5_ESM.zip › Figure_4_Source_Data/Figure_4_ABF.tiff]

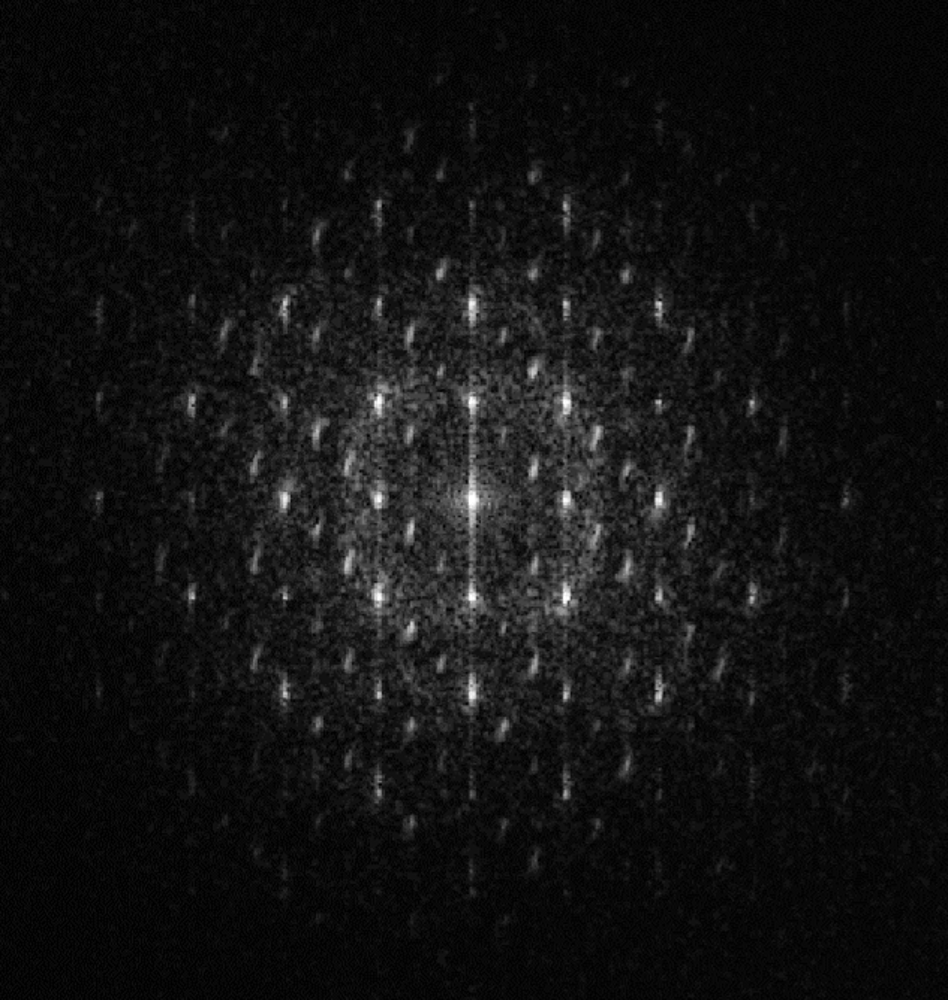

Supplement: Supplementary file 5 — Source data (TIFF files) for Fig. 4. [file 41563_2024_1797_MOESM5_ESM.zip › Figure_4_Source_Data/Figure_4_FFT.tiff]

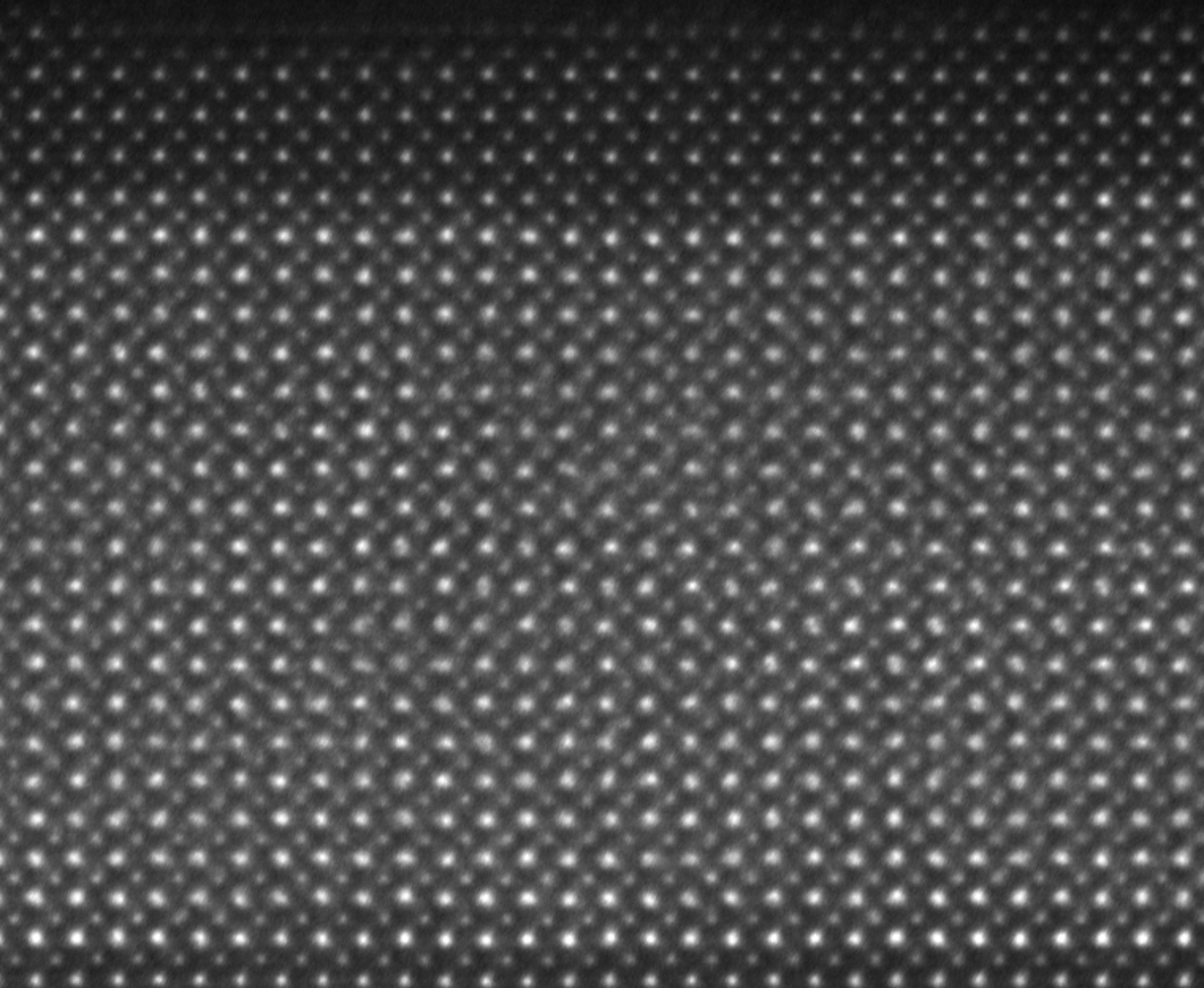

Supplement: Supplementary file 5 — Source data (TIFF files) for Fig. 4. [file 41563_2024_1797_MOESM5_ESM.zip › Figure_4_Source_Data/Figure_4_HAADF.tiff]
